# A Novel 2,7-Naphthyridine Derivative Suppresses Ovarian Cancer Cell Growth Through G1 Cell Cycle Arrest, Apoptosis Induction and Migration Inhibition

**DOI:** 10.3390/ph19101562

**Published:** 2026-10-01

**Authors:** Gabriela Chabowska, Konrad Chabowski, Helena Moreira, Anna Wójcicka, Anna Szyjka, Patrycja Koruba, Dorota Bęben, Tomasz Piasecki, Ewa Barg

**Affiliations:** 1 Department of Basic Medical Sciences, Faculty of Pharmacy, Wroclaw Medical University, Borowska Street 211, 50-556 Wroclaw, Poland; helena.moreira@umw.edu.pl (H.M.); anna.szyjka@umw.edu.pl (A.S.); dorota.beben@student.umw.edu.pl (D.B.); 2 Independent Researcher, 52-131 Wroclaw, Poland; konrad.chabowski@gmail.com; 3 Department of Organic Chemistry and Pharmaceutical Technology, Faculty of Pharmacy, Wroclaw Medical University, Borowska 211A, 50-556 Wroclaw, Poland; anna.wojcicka@umw.edu.pl; 4 Faculty of Pharmacy, Wroclaw Medical University, Borowska Street 211, 50-556 Wroclaw, Poland; patrycja.koruba@student.umw.edu.pl; 5 Department of Nanometrology, Wroclaw University of Science and Technology, Z. Janiszewskiego 11/17, 50-372 Wroclaw, Poland; tomasz.piasecki@pwr.edu.pl

**Keywords:** apoptosis, cell cycle arrest, cisplatin, electrochemical impedance spectroscopy, migration, ovarian cancer, spheroids, 2,7-naphthyridines

## Abstract

**Background/Objectives**: Ovarian cancer remains one of the deadliest gynecological malignancies due to late diagnosis, frequent recurrence, and the development of resistance to platinum-based chemotherapy. Naphthyridines constitute a diverse class of heterocyclic compounds with promising anticancer properties. The aim of this study was to evaluate the anticancer activity of three structurally related 2,7-naphthyridine derivatives (Naph-1, Naph-2, and Naph-3), identify the most active compound, and investigate its mechanism of action in ovarian cancer models. **Methods**: Cytotoxic activity was evaluated using the PrestoBlue assay in human cancer cell lines IGROV-1, MDAH-2774, SKOV-3, K562, MCF-7, and A549 and a non-cancerous CHO-K1 cell line. The most active derivative, Naph-3, was subsequently characterized in the two most sensitive ovarian cancer models (IGROV-1 and MDAH-2774) by evaluating its effects on 3D spheroid viability, cell proliferation, migration, apoptosis, and cell cycle progression. Electrochemical impedance spectroscopy was used as a label-free method for real-time monitoring of treatment-induced cellular responses. Cisplatin served as the reference compound. **Results**: Among the tested derivatives, Naph-3 demonstrated the strongest anticancer activity, with the highest sensitivity observed in ovarian cancer cell lines IGROV-1 (IC_50_ 30.31 µM) and MDAH-2774 (IC_50_ 53.68 µM). Naph-3 inhibited cell proliferation and migration, induced G1-phase cell cycle arrest, and promoted apoptotic cell death in both IGROV-1 and MDAH-2774 cells. In 3D spheroid models, Naph-3 retained significant activity in IGROV-1 spheroids, whereas MDAH-2774 spheroids displayed pronounced resistance to treatment. Although Naph-3 was less potent than cisplatin, it exhibited greater selectivity toward cancer cells, with lower toxicity against non-cancerous CHO-K1 cells. Impedance spectroscopy in a wide frequency range provided complementary kinetic information, supporting the interpretation that Naph-3 exerts a predominantly cytostatic effect, whereas cisplatin induces a more pronounced cytotoxic response. **Conclusions**: Naph-3 emerged as the most promising derivative among the tested compounds and exhibited multifaceted anticancer activity in IGROV-1 and MDAH-2774 ovarian cancer models. Its favorable selectivity profile, combined with antiproliferative, pro-apoptotic, and anti-migratory properties, highlights its potential as a promising compound for the development of novel therapeutic strategies against ovarian cancer.

## 1. Introduction

According to the American Cancer Society, ovarian cancer, named by oncologists “silent killer”, ranks sixth in cancer deaths among women and accounts for more deaths than any other gynecological tumor [1]. Generally, ovarian cancer is diagnosed mostly in postmenopausal women, but the disease also affects women even in their twenties, especially with a genetic predisposition. The early stage of the disease is usually asymptomatic, and the late-phase manifestation is often nonspecific and commonly misdiagnosed with digestive system disorders. Despite efforts made by researchers, there is no effective screening system to identify ovarian cancer at an early stage [2]. An algorithm based on annual serial CA-125 measurements followed by transvaginal ultrasonography is recommended only for women at increased risk, especially with *BRCA1*, *BRCA2*, and Lynch syndrome gene mutations. On the other hand, 90% of patients suffer from non-hereditary ovarian cancer. All these factors cause more than 75% of affected women to be diagnosed at an advanced stage of illness [3].

**Figure 1:**
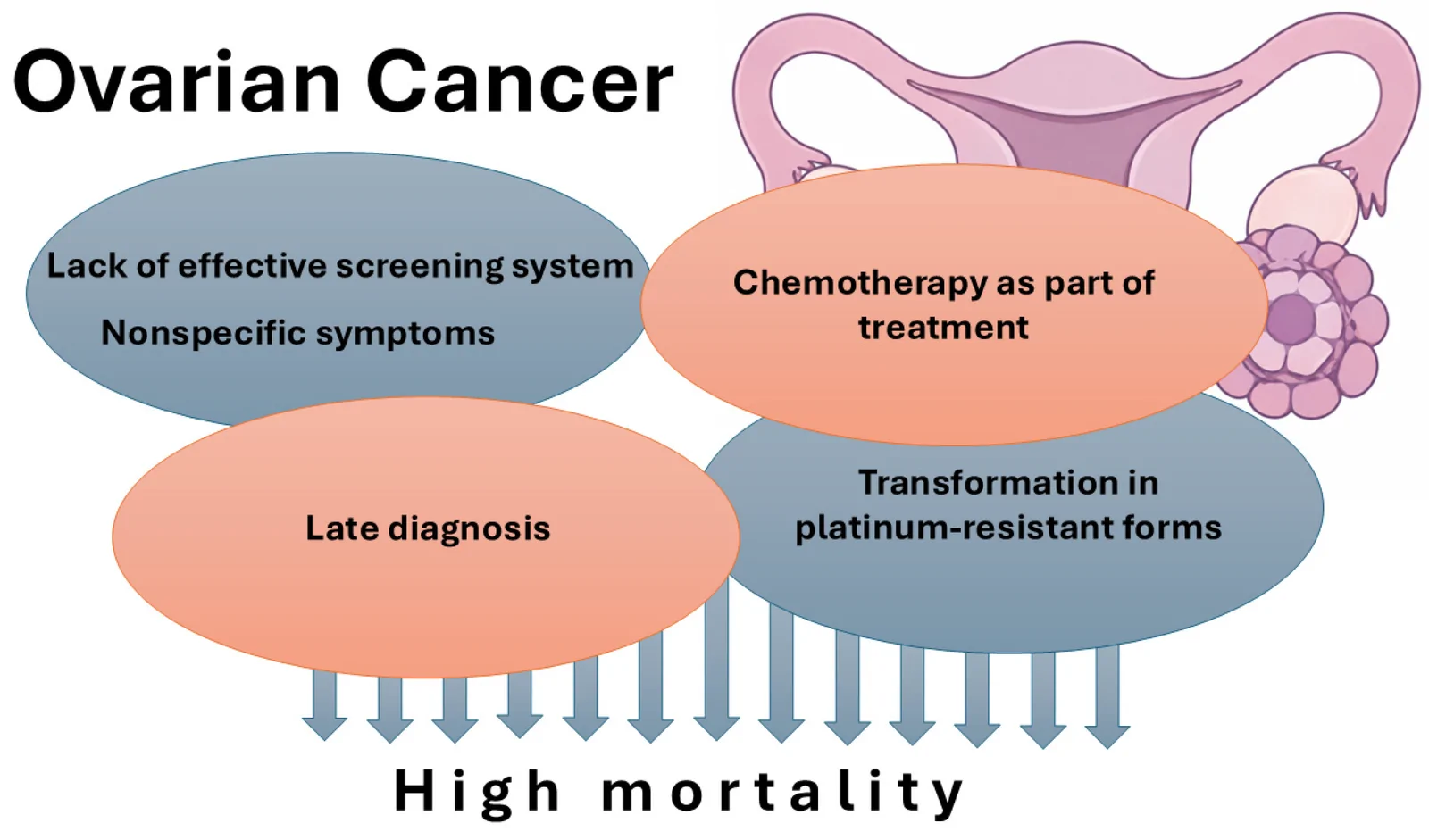
Factors influencing low survival rates among patients with stage III and IV ovarian cancer.

Despite advances in targeted therapies, chemotherapy still plays an essential role in late-stage ovarian cancer treatment and is administered to the majority of patients. It is used as a neoadjuvant or post-adjuvant course before or after surgical procedures. The use of presurgical chemotherapy allows for cytoreductive surgery in patients at a late stage of the disease. Postoperative chemotherapy aims to reduce tumor remnants and metastases and decrease the risk of relapse. It can be administered intraperitoneally, intravenously, or in combination [4]. First-line chemotherapy regimens include a combination of platinum agents and paclitaxel. Despite high initial effectiveness, most commonly diagnosed ovarian carcinomas frequently relapse into platinum-resistant forms, with 5-year survival rates remaining below 40% in patients with advanced-stage disease (Figure 1) [5]. Current strategies to overcome therapeutic resistance in ovarian cancer increasingly focus on targeting the molecular mechanisms underlying treatment failure. These approaches include modulation of drug influx and efflux (use of P-glycoprotein inhibitors, administration of antibody–drug conjugates, and the therapeutic application of non-coding RNAs), targeting DNA damage repair mechanisms (inhibition of ATR/ATM kinases, WEE1, and CHK1/2), inhibition of pro-survival signaling pathways (PI3K/AKT, GAS6-AXL, MAPK, Notch, and NF-κB), and modulation of epigenetic mechanisms (coadministration of hypomethylating agents) [6]. Recent studies have also highlighted the important role of the tumor microenvironment in platinum resistance. In particular, neutrophil extracellular traps (NETs) have been shown to promote cisplatin resistance through TGF-β/Smad2-mediated activation of SHP-1, suggesting that targeting the NETs/SHP-1 axis may represent a potential strategy for restoring platinum sensitivity in ovarian cancer [7]. Beyond approaches aimed at reversing specific resistance mechanisms, increasing attention is being directed toward the development of novel compounds acting through alternative molecular targets. For example, SIRT2-targeting PROTAC degraders have recently demonstrated antitumor activity in ovarian cancer models through modulation of the SIRT2/AKT/mTOR signaling pathway [8]. These advances highlight the continued importance of identifying novel chemical scaffolds with distinct anticancer properties. In this context, naphthyridine derivatives have attracted considerable interest because of their structural diversity and broad spectrum of biological activities, making them promising candidates for developing novel anticancer agents.

Naphthyridines are bioactive heterocyclic compounds, called also in the chemical literature as “diazanaphthalenes”, according to possessing a fused system of two pyridine rings. The Naphthyridine group comprises six positional isomers, depending on the locations of nitrogen atoms: 1,5-, 1,6-, 1,7-, 1,8-, 2,6-, and 2,7-naphthyridines. The compounds can be obtained both from natural sources and from chemical synthesis. Naphthyridines remain interesting compounds due to their multimodal activity, including anticancer potency [9]. The anticancer potential of naphthyridine derivatives has been extensively investigated in recent years. Numerous representatives of this heterocyclic family have demonstrated significant cytotoxic activity against a broad spectrum of human malignancies, including lung, breast, prostate, cervical, liver, and hematological cancers [9,10,11,12]. Some representatives of the group were investigated further and presented diverse anticancer mechanisms. Cystodytine J, Diplamine, Shermilamines B and C, and Eilatin were shown to inhibit topoisomerase II and intercalate into DNA [13]. Canthin-6-one was reported to activate apoptosis and necrosis in Kasumi-1human myeloid leukemia cells, inducing cell cycle arrest at G0/G1 and G2 phases at 7 μM and 45 μM, respectively, and stimulating cancer cells differentiation [14]. Aaptamine arrested cell cycle at the G1 phase via selectively impairing of cell cycle regulation drivers (CDK2/4 and Cyclin D1/E), and inhibited proproliferative PI3K/AKT/GSK3b axis in non-small cell lung cancer in vitro. It also decreases expression levels of prometastatic MMP9 and MMP7 [15]. Bisleuconothine A, a bisindole alkaloid, inhibited WNT signaling and induced apoptosis by increasing the cleavage of caspases [16]. Aaptamine activated p21 promoter in a p53-independent manner [17]. The compound was also reported to induce cell cycle arrest via increase in p21 binding to Cdk2-cyclin D/E complexes [18]. Naphthyridines were shown to be apoptosis inducers via p53-dependent pathway [19]. Lissoclinidine B inhibited ubiquitylation and stabilized proapoptotic p53 protein [20]. Matrine exerted anticancer effects by decreasing cell viability and promotion of apoptosis through AKT/mTOR axis in breast cancer cell line [21]. The compound was effective in both in vitro and in vivo models [22]. Furthermore, matrine suppressed ABCB1 drug transport, which play a key role in multidrug resistance effect [23]. Importantly, increasing evidence supports the relevance of naphthyridines in ovarian cancer research. Several naturally occurring and synthetic naphthyridine compounds have demonstrated promising activity in ovarian cancer models, including the induction of apoptosis, suppression of proliferation, and inhibition of metastatic behavior [22,24,25]. Furthermore, the 1,8-naphthyridine derivative vosaroxin advanced to clinical evaluation in platinum-resistant ovarian cancer, highlighting the translational potential of this class of compounds [26]. These findings indicate that naphthyridines represent a promising scaffold for the development of novel therapeutic agents against ovarian cancer.

**Figure 2:**
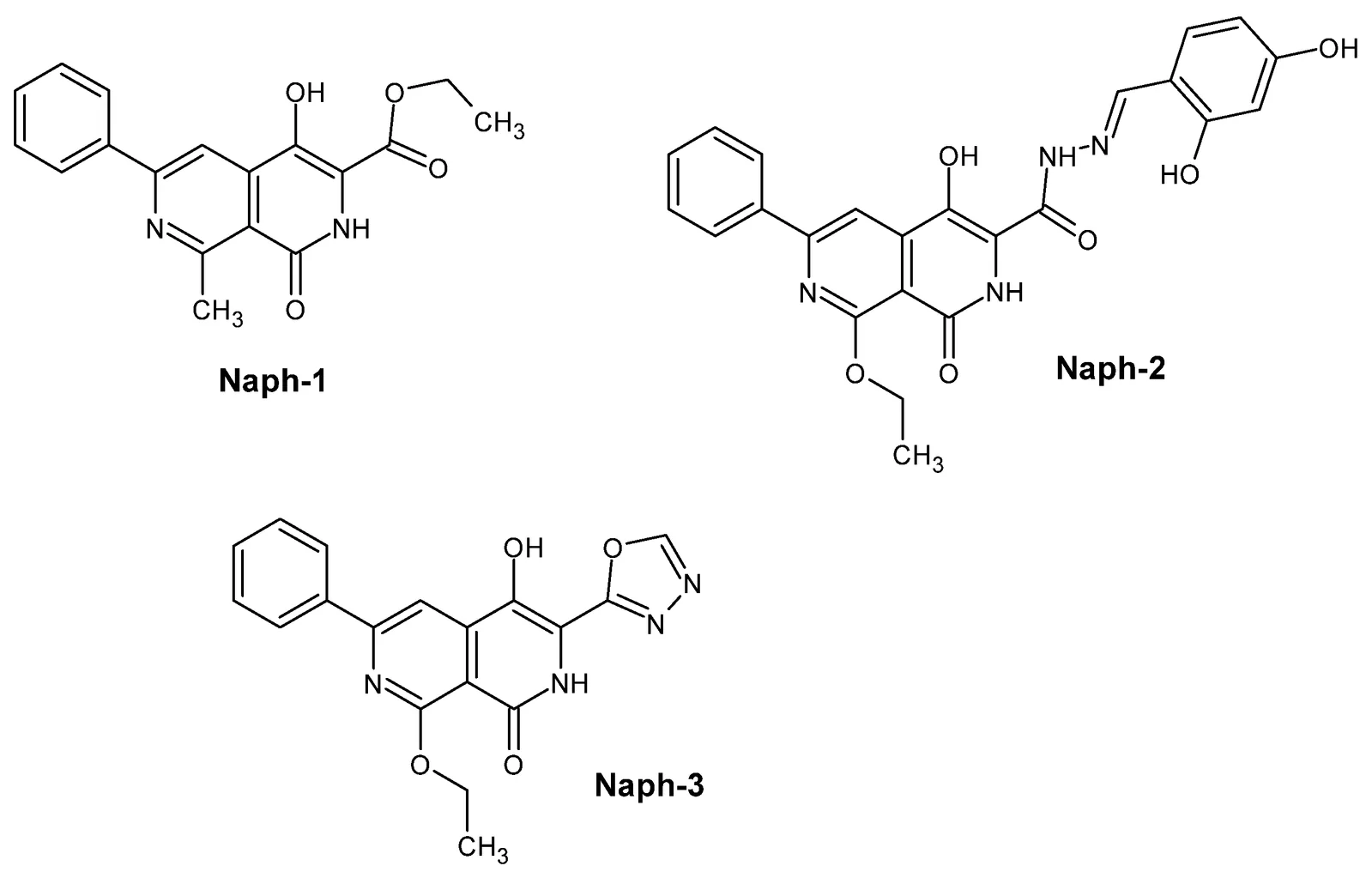
2,7-Naphthyridine derivatives-structures.

2,7-naphthyridine derivatives were synthesized by Anna Wójcicka et al. at Wroclaw Medical University [27,28]. The compounds were screened in vitro against 60 different human tumor cell lines at National Cancer Institute NCI (Bethesda, MD, USA) representing nine cancer diseases: leukemia, melanoma, cancers of the breast, lung, brain, colon, prostate, ovary, renal. Derivatives Naph-1, Naph-2, and Naph-3 exerted a promising cytostatic effect on a wide panel of cancer cell lines, including drug-resistant ovarian cancer cells SKOV-3 (Figure 2). Growth inhibition rates were 84.39% (IGROV-1) and 58.38% (SKOV-3) for Naph-1, 98.57% (IGROV-1) for Naph-2, and 63.27% (IGROV-1) and 40.67% (SKOV-3) for Naph-3 [27,28]. The compounds Naph-1 and Naph-3 were patented in 2014 and 2017 (nr 215840 and 227264, respectively). Based on their promising activity profiles against ovarian cancer in the NCI screening, Naph-1, Naph-2, and Naph-3 were selected for further, more comprehensive biological characterization in the present study. Therefore, the aim of the present study was to evaluate the anticancer potential of three structurally related 2,7-naphthyridine derivatives—Naph-1, Naph-2, and Naph-3—against a panel of human cancer cell lines and to identify the most promising compound. Comparative analysis of the tested derivatives was also performed to provide preliminary insights into the structure–activity relationships (SAR) underlying their biological activity. Following the initial screening, the mechanism of action of the most active derivative was investigated in ovarian cancer models through the assessment of cell viability, proliferation, migration, cell cycle progression, apoptosis induction, and growth in 3D spheroid cultures. Furthermore, electrochemical impedance spectroscopy was used as a label-free, real-time approach to monitor treatment-induced alterations in cellular behaviour. Throughout the study, cisplatin, the current standard of care in ovarian cancer chemotherapy, served as the reference compound.

## 2. Results

### 2.1. Chemical Synthesis

**Figure 3:**
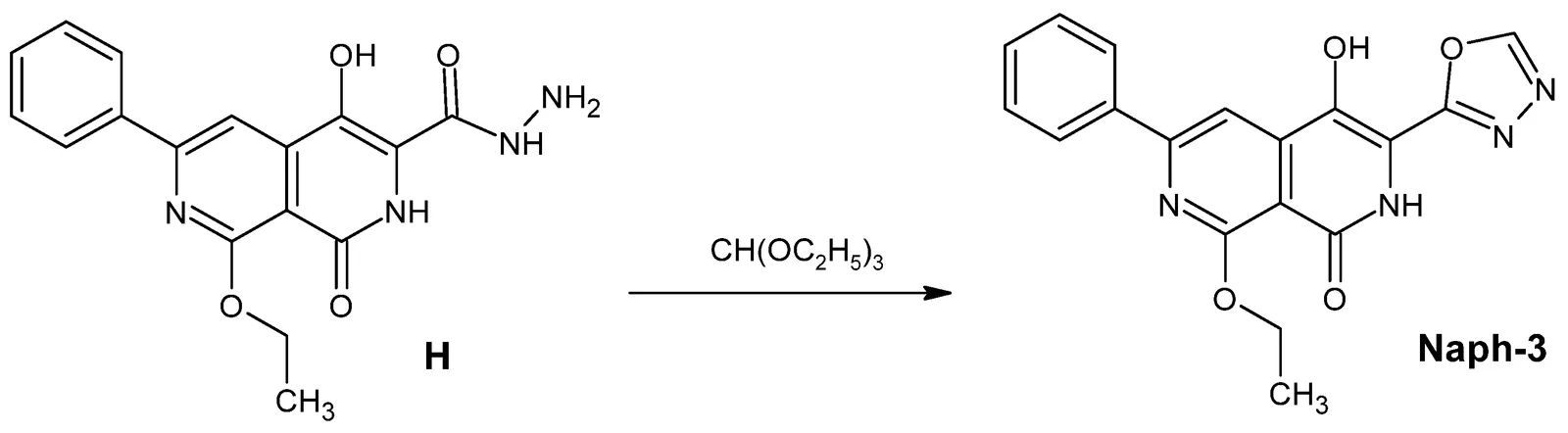
Synthesis of 8-ethoxy-4-hydroxy-3-(1,3,4-oxadiazol-2-yl)-6-phenyl-2,7-naphthyridin-1(2*H*)-one Naph-3.

As a continuation of our studies on the biological activity of 2,7-naphthyridines, the compounds obtained in previous years (Figure 2) were evaluated for their antiproliferative properties [27,29]. The synthesis and characterization of derivatives Naph-1 and Naph-2 have been published previously [27,29]. 8-Ethoxy-4-hydroxy-3-(1,3,4-oxadiazol-2-yl)-6-phenyl-2,7-naphthyridin-1(2*H*)-one Naph-3 was synthesized according to the procedure described in the patent [30]. Cyclocondensation of the previously obtained 8-ethoxy-4-hydroxy-1-oxo-6-phenyl-1,2-dihydro-2,7-naphthyridine-3-carbohydrazide **H** [28] with triethyl orthoformate led to the formation of Naph-3 (Figure 3). Spectral analyses of new compound Naph-3 are shown in the Supplementary Material (Figures S1–S3).

### 2.2. Cell Viability Evaluation

In this study, cell viability was determined using the PrestoBlue Assay, which measures the metabolic activity of cells. The effects of naphthyridine derivatives on cell viability were evaluated following 72 h of incubation with the tested compounds and assessed relative to untreated cell cultures control (E/E_0_). The obtained results were presented as IC_50_ (half-maximal inhibitory concentration) values, indicating the concentration required to inhibit 50% of cell viability (Table 1).

Among the tested compounds, Naph-3 exhibited the most pronounced biological activity. This derivative demonstrated cell viability reducing effects across nearly all evaluated cancer cell lines, with particularly strong activity against ovarian cancer models IGROV-1 (IC_50_ = 30.31 µM) and MDAH-2774 (IC_50_ = 53.68 µM), as well as the leukemia cell line K562 (IC_50_ = 50.52 µM). Notably, the IC_50_ value in the non-cancerous CHO-K1 cell line was significantly higher (IC_50_ = 77.19 µM) compared to those obtained for the above cancer cell lines, indicating a degree of selectivity toward malignant cells. In the other cell lines, Naph-3 showed modest cytotoxic activity, with IC_50_ values of 159.17 µM and 180.31 µM for MCF-7 and A549, and exceeding 200 µM in the SKOV-3.

Naph-1 demonstrated limited cytotoxic activity. However, a measurable cell viability reduction was observed in selected cancer cell lines: ovarian MDAH-2774 (IC_50_ = 109.06 µM), SKOV3 (IC_50_ = 161.43 µM), and breast MCF-7 (IC_50_ = 171.06 µM). No toxicity was observed in non cancer CHO-K1 cells (IC_50_ value above 200 µM). For other cancer cell models (IGROV-1, A549, K562), IC_50_ values exceed 200 µM, indicating low activity within the tested concentration range.

Naph-2 showed the lowest potential to reduce cancer cell viability, with measurable effects observed only in the ovarian cancer cells MDAH-2774 (IC_50_ = 152.90 µM). In the other evaluated cell lines, the compound showed activity with IC_50_ values exceeding 200 µM.

Cisplatin, used as a reference drug, exhibited the highest cytotoxic effect across all tested cell lines, with IC_50_ values ranging from 7.92 µM (SKOV3) to 46.42 µM (MCF-7). However, in the non-cancerous CHO-K1 cell line, the IC_50_ of 10.50 µM indicate low selectivity and consistent with its well-known toxicity profile.

Based on the results of the initial cytotoxicity screening, Naph-3 was selected for further studies as the most active compound among the tested naphthyridines. Ovarian cancer models, IGROV-1 and MDAH-2774, the most sensitive to Naph-3 cytotoxic properties, were selected to investigate the underlying mechanisms of its activity. In all experimental stages, the effect was compared to cisplatin, reference drug used as golden standard in ovarian chemotherapy regimens. The agents were tested in three concentrations: approximately corresponding and above IC_50_ values.

### 2.3. Cell Viability Evaluation in Spheroid 3D Models

**Figure 4:**
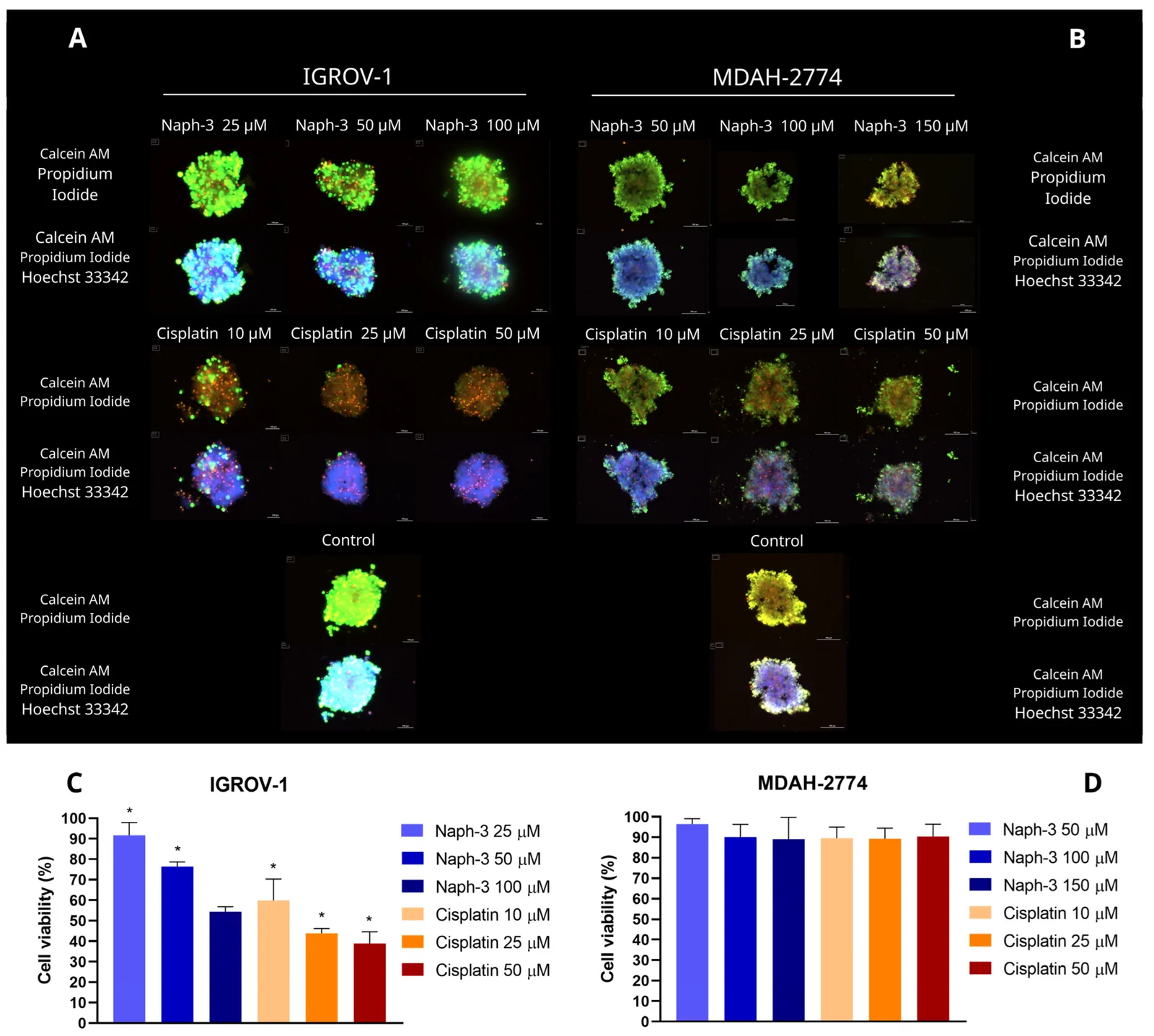
Effect of Naph-3 and cisplatin on the viability of ovarian cancer spheroids. Representative fluorescence images of IGROV-1 (**A**) and MDAH-2774 (**B**) spheroids after 72 h of treatment with Naph-3 or cisplatin at the indicated concentrations. Live cells are stained green (Calcein AM), whereas dead cells are stained red (Propidium Iodide); nuclei are counterstained blue (Hoechst 33342). (**C**,**D**) Quantitative analysis of cell viability in IGROV-1 (**C**) and MDAH-2774 (**D**) spheroids compared to the control spheroid culture untreated with Naph-3 nor cisplatin. Cell viability was defined as the ratio of the total fluorescence intensity in the GFP channel (Calcein AM-positive viable cells) to the sum of the total fluorescence intensities in the GFP and RFP channels (Calcein AM- and Propidium Iodide-positive cells). Data are expressed relative to the untreated control (set to 100%). Data are presented as mean ± SD from three independent experiments (*n* = 3). Statistical significance was assessed using one-way ANOVA followed by Dunnett’s multiple comparisons test (* *p* < 0.05) versus the untreated control group.

Three-dimensional (3D) spheroid cultures are considered more physiologically relevant tumor models than conventional monolayer cultures, as they better mimic the architecture and microenvironment of solid tumors. Spheroids reproduce key features of in vivo tumors, including cell–cell interactions, nutrient and oxygen gradients, and barriers to drug penetration, which may influence therapeutic response. Therefore, the effects of Naph-3 and cisplatin on cell viability were additionally evaluated in IGROV-1 and MDAH-2774 ovarian cancer spheroids. Cells viability was assessed using the Live Dead Cell Viability Assay Kit for 3D and 2D Cell Culture (Hoechst 33342, Propidium Iodide, and Calcein-AM), enabling simultaneous visualization and quantification of total, dead, and viable cell populations, respectively (Figure 4).

In IGROV-1 spheroid models, both Naph-3 and cisplatin exhibited pronounced cytotoxic activity. The strongest effect was observed for cisplatin at 50 µM, which reduced cell viability to 38.9% of the control value. A comparable reduction in viability was achieved with cisplatin at 25 µM (43.9%) and Naph-3 at 100 µM (54.4%). Naph-3 demonstrated a concentration-dependent effect, with cell viability decreasing from 91.7% at 25 µM to 54.4% at 100 µM. These findings indicate that IGROV-1 spheroids retained considerable sensitivity to both the reference drug and the tested compound, with the highest concentration of Naph-3 exhibiting activity comparable to that of cisplatin.

A markedly different response was observed in MDAH-2774 spheroids. Regardless of the compound or concentration applied, cell viability remained high, ranging from 89.1% to 96.5% of the control. No clear concentration-dependent effect was detected for either Naph-3 or cisplatin. Even the highest tested concentrations of both agents failed to induce a substantial reduction in cell viability, indicating limited cytotoxic activity in this 3D model.

### 2.4. Scratch Assay

Cancer cell migration is a critical process in tumour progression and metastasis, driven by coordinated changes in cell adhesion, cytoskeletal organization, and signalling pathways, including epithelial–mesenchymal transition. Consequently, targeting cell migration represents a promising therapeutic strategy to limit metastatic spread and improve cancer treatment outcomes.

**Figure 5:**
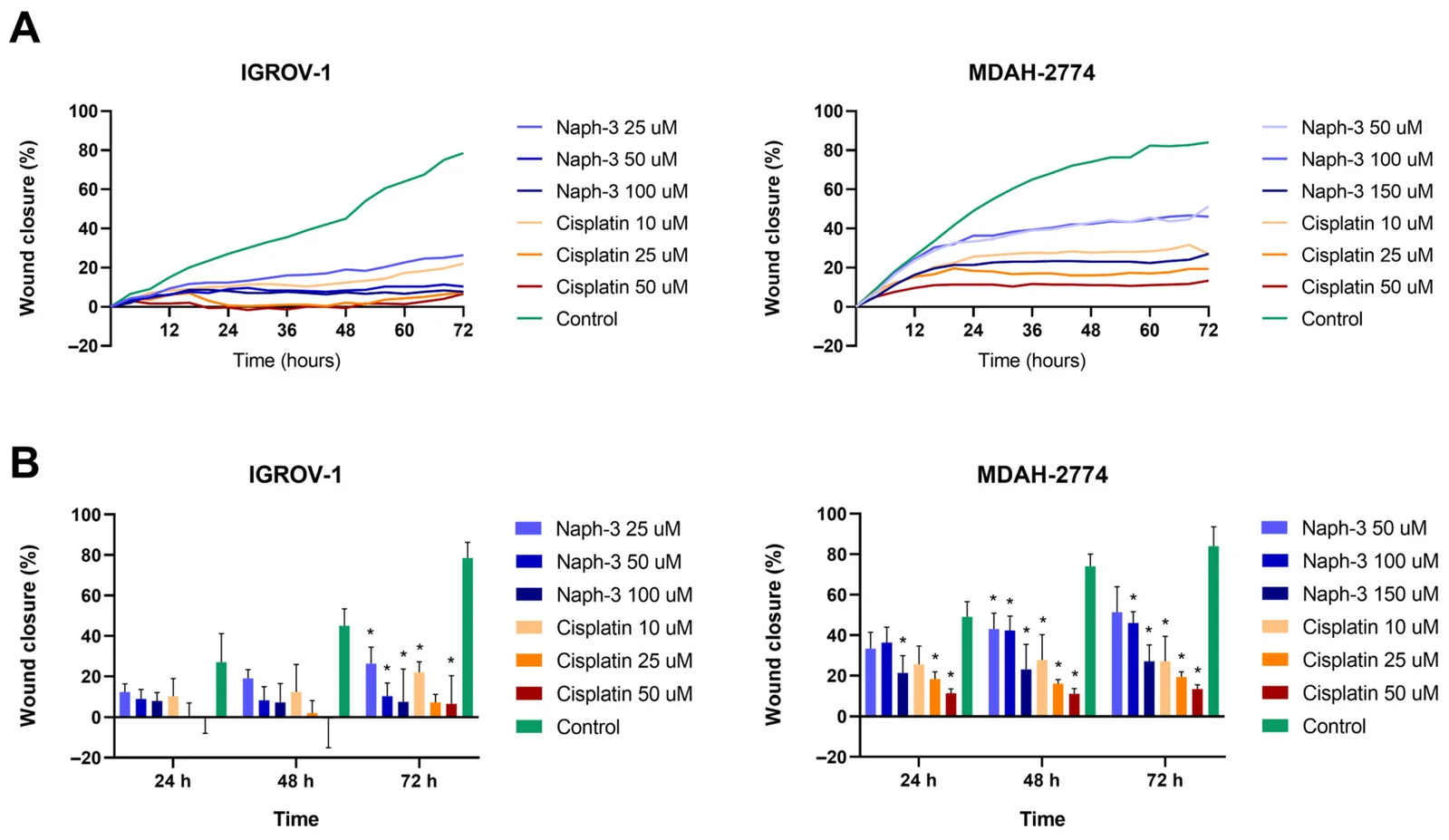
Effect of Naph-3 and cisplatin on wound closure of ovarian cancer cells determined by the wound healing assay. (**A**) Representative quantification of wound closure in IGROV-1 and MDAH-2774 cells treated with Naph-3 or cisplatin over a 72 h incubation period. (**B**) Wound closure at selected time points expressed as a percentage of untreated cell culture control. Data are presented as mean ± SD from three independent experiments (*n* = 3). Statistical significance was assessed using Two-way repeated measures ANOVA followed by Dunnett’s multiple comparisons test (* *p* < 0.05) versus the untreated control group.

To assess the impact of Naph-3 on cancer cell migration, a scratch wound assay was conducted using the Lionheart FX imaging system in IGROV-1 and MDAH-2774 ovarian cancer cell lines. Inhibition of cell migration was assessed by monitoring the scratch closure over 72 h of incubation with the tested compounds (Figure 5).

Reduced wound closure in Naph-3- and cisplatin-treated cells compared with untreated controls was already apparent within the first 24 h of the assay, when cell proliferation is expected to contribute less to reduction in scratch area. In IGROV-1 cells, wound closure after 24 h reached 27.0% in the control group, whereas exposure to Naph-3 resulted in closure values of 12.3%, 9.0%, and 8.0% at 25, 50, and 100 µM, respectively. Cisplatin-treated cells showed wound closure of 10.3%, 0.7%, and −0.3% at 10, 25, and 50 µM, respectively. None of the results obtained for the tested compounds reached statistical significance.

In MDAH-2774 cells, wound closure proceeded more rapidly, reaching 49.0% in untreated cells within the first 24 h. In comparison, wound closure was 33.3%, 36.3%, and 21.3% following treatment with 50, 100, and 150 µM Naph-3, respectively. Cisplatin treatment resulted in closure values of 25.7%, 18.3%, and 11.3% at 10, 25, and 50 µM, respectively. Statistical significance compared with the control was reached for Naph-3 at 150 µM and for cisplatin at 25 and 50 µM.

With prolonged incubation period, the IGROV-1 cell line demonstrated higher sensitivity to treatment. Compared to the untreated control, which reached 78.5% wound closure after 72 h, both Naph-3 and the reference drug cisplatin significantly reduced wound closure to below 24% at all tested concentrations. The observed inhibitory effect, relative to the control, was evident throughout the all incubation period. In addition, exposition to Naph-3 resulted in a dose-dependent inhibitory effect. At 25 µM, partial inhibition was observed (26.3% closure at 72 h), whereas higher concentrations 50 µM and 100 µM markedly reduced wound closure to 10.3% and 7.7%. Cisplatin also effectively reduced wound closure in dose dependent manner with results of 22.0%, 7.3% and 6.7% for concentrations 10, 25 and 50 µM. Importantly, no statistically significant differences were observed between the effects of Naph-3 and cisplatin throughout the entire incubation period of the cells with the tested compounds.

In MDAH-2774 cells, the untreated control group demonstrated 84.0% wound closure after 72 h. However, these cells exhibited reduced sensitivity to both treatments. Naph-3 showed a concentration-dependent effect, although less pronounced than in IGROV-1 cells. The highest concentration 150 µM reduced finally wound closure to 27,0%, while lower concentrations of 50 and 100 µM resulted in moderate inhibition of 46.0% and 51.3%. Cisplatin exhibited mainly a statistically significantly greater effect, with wound closure values of 27.0%, 19.3%, and 13.3% at concentrations of 10 µM, 25 µM, and 50 µM, respectively. No statistically significant differences were observed in MDAH-2774 wound closure between cells treated with Naph-3 at the highest concentration (150 µM) and those treated with any of the tested concentrations of cisplatin. Likewise, no significant differences were found between Naph-3 at 100 or 50 µM and cisplatin at 10 µM. However, prolonged incubation period has strict limitations: cannot distinguish clearly between migration and proliferation.

In conclusion, these findings indicate that Naph-3 exhibits anti-migratory activity comparable to cisplatin at selected concentrations, suggesting similar efficacy in inhibiting cancer cell migration under the tested conditions. However, given the inherent limitations of the scratch assay, particularly the contribution of cell proliferation to scratch closure, the results should be interpreted as preliminary evidence rather than a direct measure of migration.

### 2.5. Confluence Evaluation

Uncontrolled cell proliferation is one of the hallmarks of cancer and plays a crucial role in carcinogenesis. Therefore, cytostatic activity is considered an important therapeutic strategy in the development of novel anticancer agents.

**Figure 6:**
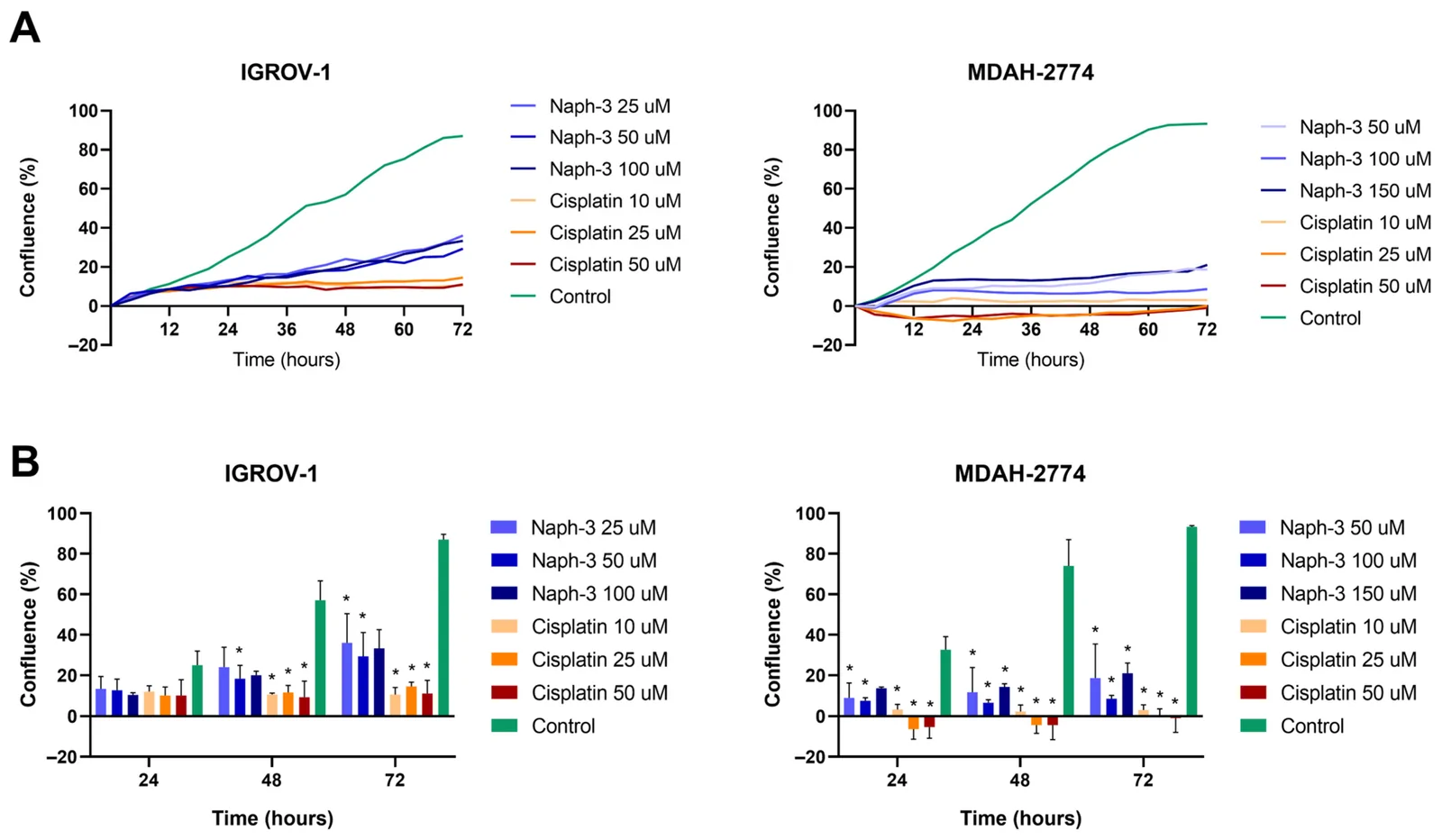
Effect of Naph-3 and cisplatin on the proliferation of ovarian cancer cells. (**A**) Time-dependent changes in the confluence of IGROV-1 and MDAH-2774 cells treated with Naph-3 or cisplatin monitored using the Lionheart FX live-cell imaging system over a 72 h incubation period. Confluence values are presented as changes relative to the initial measurement (0 h), with baseline values normalized to zero. (**B**) Relative cell confluence at selected time points. Data are presented as mean ± SD from three independent experiments (*n* = 3). Statistical significance was assessed using Two-way repeated measures ANOVA followed by Dunnett’s multiple comparisons test (* *p* < 0.05) versus the untreated control group.

The antiproliferative effects of Naph-3 and cisplatin were assessed by real-time monitoring Lionheart FX imaging system and analyzed as percentage of cell confluence. The cells were monitored for 72 h (Figure 6).

In IGROV-1, control cells exhibited continuous and rapid proliferation, reaching approximately 87.0% confluence at the end of the experiment, indicating normal growth under untreated conditions. Exposition to Naph-3 at concentrations of 25, 50, and 100 µM significantly reduced cell proliferation to 36.0%, 29.3%, and 33.3%, respectively. No significant concentration-dependent effect was observed. The effect of the reference cisplatin was stronger and concentration-independent, with cell confluence remaining stable within the range of 9.0–14.5% from 24 h onward, most likely due to the strong cytotoxic activity of the drug. At the final time point (72 h), treatment with Naph-3 significantly reduced cell confluence in IGROV-1 cells at concentrations of 50 μM (*p* = 0.046) and 100 μM (*p* = 0.028), compared with the untreated control, whereas the effect of 25 μM did not reach statistical significance (*p* = 0.092). Cisplatin significantly decreased cell confluence at all tested concentrations (10–50 μM; *p* ≤ 0.01). No statistically significant differences were observed among the tested concentrations of Naph-3 or cisplatin, nor between the effects of Naph-3 and cisplatin.

In MDAH-2774 cell line, compared with the untreated control, all tested compounds markedly inhibited cell growth. In Naph-3-treated cells, confluence did not exceed approximately 21.0% after 72 h, confirming a substantial reduction in proliferative capacity. Treatment with Naph-3 at concentration of 100 µM maintained cell confluence below 8.7% for most of the experiment, whereas the 50 and 150 µM concentrations resulted in a gradual increase to 18.7% and 21.0%.

The reference cisplatin demonstrated stronger antiproliferative activity, with cell confluence remaining close to 0.0% throughout the entire experiment regardless of concentration. This effect was most likely associated with the pronounced cytotoxic activity of the compound.

At the final timepoint of 72 h, a statistically significant reduction in confluence compared with the untreated control was observed after treatment with 100 μM (*p* < 0.0001) and 150 μM (*p* = 0.006), whereas the effect of 50 μM did not reach statistical significance (*p* = 0.0643). Cisplatin significantly reduced cell confluence at all tested concentrations (10–50 μM; *p* ≤ 0.008). No significant differences were detected among the tested concentrations of Naph-3 or cisplatin. Likewise, no significant differences were observed between Naph-3 and cisplatin across the tested concentration range, with the exception of Naph-3 at 150 μM, which differed significantly from cisplatin at 25 μM (*p* = 0.034).

Overall, the MDAH-2774 cell line demonstrated higher sensitivity to the tested compounds. While cisplatin tended to produce a stronger antiproliferative effect, the majority of pairwise comparisons between cisplatin and Naph-3 did not reveal statistically significant differences, suggesting comparable inhibition of cell proliferation by both compounds.

### 2.6. Effect on Cell Cycle Progression

Cell cycle arrest represents one of the key targets of anticancer agents, as disruption of cell cycle progression effectively inhibits tumor cell proliferation and may consequently lead to programmed cell death.

**Figure 7:**
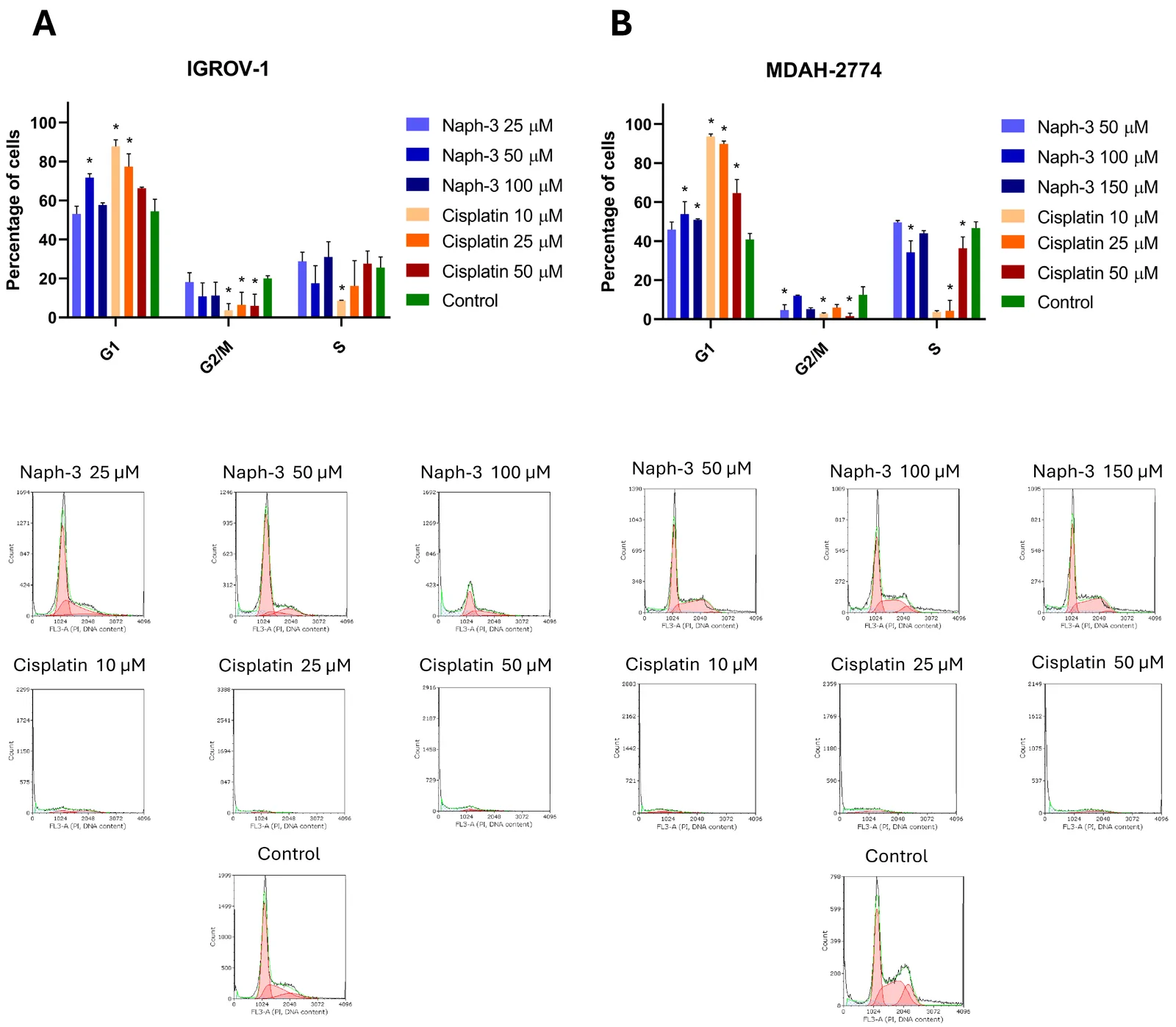
Effect of Naph-3 and cisplatin on cell cycle distribution in ovarian cancer cells. (**A**) IGROV-1 and (**B**) MDAH-2774 cells were treated with Naph-3 or cisplatin for 72 h. Cell cycle distribution was determined by flow cytometry following DNA staining with propidium iodide (PI). The upper panels show the percentages of cells in the G1, G2/M, and S phases, while the lower panels present representative DNA content histograms for each treatment group. The experimental DNA content distribution is shown in green, while the fitted cell cycle model is shown in red. Data are expressed as mean ± SD from three independent experiments (*n* = 3). Statistical significance was assessed using Two-way ANOVA followed by Dunnett’s multiple comparisons test (* *p* < 0.05) versus the untreated control group.

The effect on cell cycle after 72 h incubation with tested compounds was evaluated by flow cytometry using the FxCycle^TM^ PI/RNase Staining Solution (Figure 7).

Both, Naph-3 and the reference drug cisplatin exhibited the ability to induce cell cycle arrest in ovarian cancer models. The tested compounds led to the accumulation of cancer cells in the G1 phase, accompanied by a reduction in the percentage of cells in the S and G2/M phases. For Naph-3, the most pronounced effect was observed in the IGROV-1 cell line at a concentration of 50 µM, where the percentage of cells in the G1 phase reached 71.8%. In the MDAH-2774 cell line, the effect was lower but remained statistically significant compared with the control for concentrations of 100 µM (53.8%) and 150 µM (50.9%).

Cisplatin induced the most pronounced G1 phase arrest in the MDAH-2774 cell line across all tested concentrations, reaching 93.6%, 89.7%, and 64.5% for 10, 25, and 50 µM, respectively. In IGROV-1 cells, the effect was weaker and statistically significant at concentrations of 10 µM (87.8%) and 25 µM (77.4%). Importantly, for both compounds, the strongest inhibitory effect was not observed at the highest tested concentrations, indicating an inverse dose–response relationship rather than a strictly dose-dependent effect. This phenomenon may be explained by the greater cytotoxicity observed at higher concentrations, resulting in an increased proportion of fragmented and dead cells, as reflected by the events preceding the G1 peak in the cell cycle histograms.

Overall, both Naph-3 and cisplatin inhibited cell cycle progression in ovarian cancer cells IGROV-1 and MDAH-2774 by inducing G1 phase arrest. The obtained results suggest that disruption of cell cycle regulation may represent one of the mechanisms underlying the antiproliferative activity of the tested compounds.

### 2.7. Effect on Apoptosis Induction

**Figure 8:**
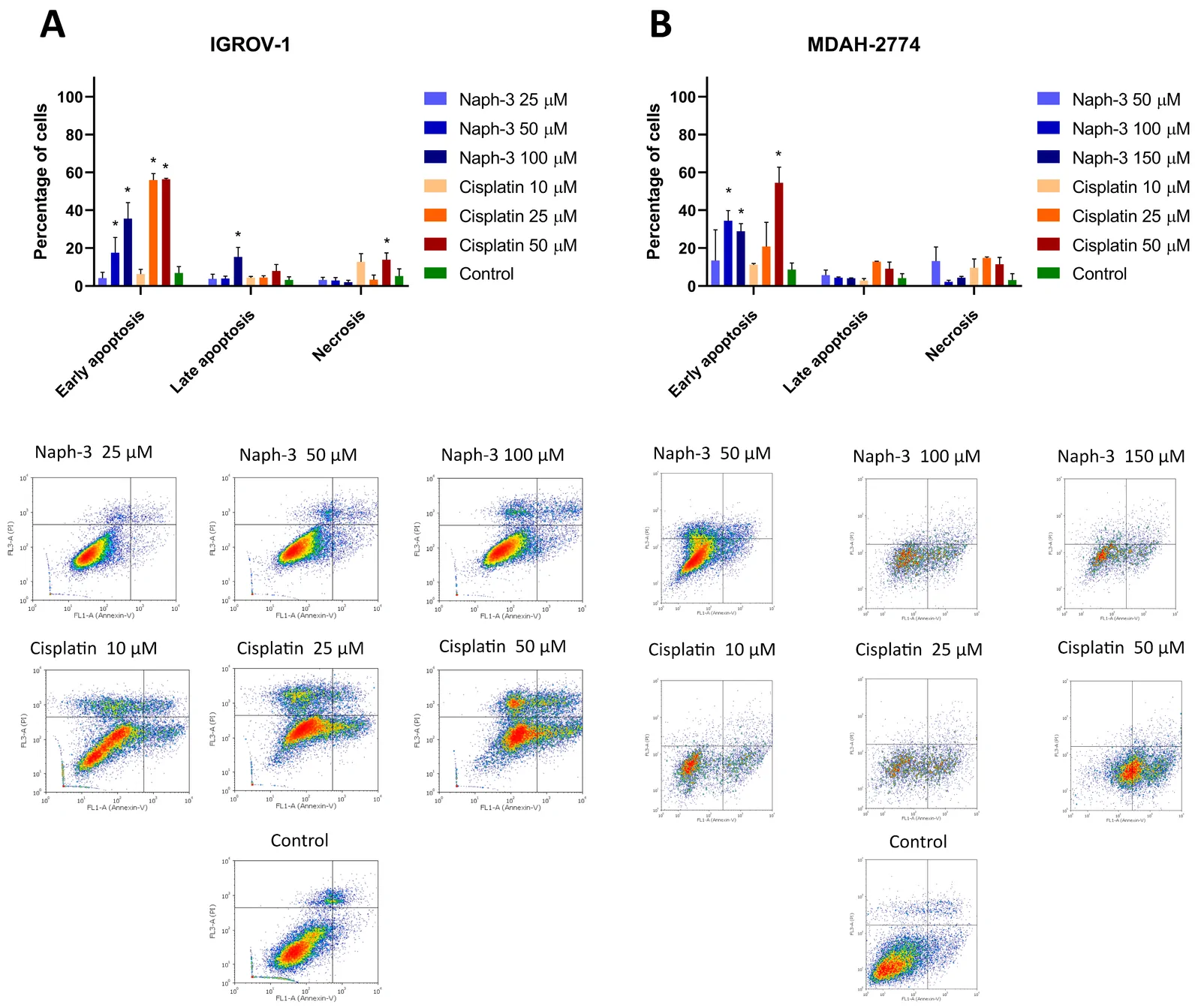
Effect of Naph-3 and cisplatin on apoptosis induction in ovarian cancer cells. (**A**) IGROV-1 cells and (**B**) MDAH-2774 cells were treated with Naph-3 or cisplatin for 72 h. Apoptosis was evaluated by flow cytometry using Annexin V-FITC/Propidium Iodide (PI) staining. Bar graphs show the percentages of cells undergoing early apoptosis, late apoptosis, and necrosis. Representative Annexin V/PI dot plots are presented below each graph. Colors in the density plots represent event density, with warmer colors indicating regions of higher event density and cooler colors indicating regions of lower event density. Data are expressed as mean ± SD from three independent experiments (*n* = 3). Statistical significance was assessed using Two-way ANOVA followed by Dunnett’s multiple comparisons test (* *p* < 0.05) versus the untreated control group.

Apoptosis, defined as programmed cell death, is mechanism responsible for the proper development, homeostasis and prevention of excessive, harmful proliferation of the body’s cells. Since the process is commonly impaired in cancer cells, the ability to restore apoptotic signaling represents a major mechanism underlying the anticancer activity of many chemotherapeutic agents. In this study, impact on cell apoptosis was evaluated by flow cytometry using Alexa Fluor 488 Annexin V/Dead Cell Apoptosis Kit (Figure 8).

Both IGROV-1 and MDAH-2774 ovarian cancer cell lines demonstrated increased apoptotic cell death following treatment with Naph-3 and cisplatin, although the magnitude of the response differed between both cell lines, cell death was predominantly associated with early apoptosis, while late apoptosis and necrosis contributed only marginally. In IGROV-1 cells, cisplatin exhibited the strongest pro-apoptotic activity, particularly at concentrations of 50 µM and 25 µM, leading to a marked elevation in the percentage (56.3% and 56.9%, respectively) of early apoptotic cells compared with the control group (6.9%). Naph-3 also induced apoptosis in a concentration-dependent manner, with the highest tested concentration 100 µM inducing a substantial increase in early apoptotic cells of 35.5%. Additionally, treatment with 100 µM Naph-3 resulted in a moderate increase in the proportion of late apoptotic cells (17.5%), indicating progression of the apoptotic process. Necrotic cell death remained relatively limited, suggesting that the tested compounds primarily activated programmed cell death pathways rather than causing nonspecific cytotoxicity.

Similarly, in the MDAH-2774 cell line, cisplatin induced the most pronounced apoptotic response, especially at 50 µM, where the percentage of early apoptotic cells reached 54.49% compared with control group (8.7%). Naph-3 treatment also significantly increased the proportion of apoptotic cells, especially at concentrations of 100 and 150 µM (34.5% and 28.9%, respectively), although the effect was less pronounced than that observed with cisplatin. In contrast to IGROV-1 cells, MDAH-2774 cells displayed slightly higher levels of necrosis following treatment with Naph-3 and cisplatin, however the effect was not significative. This may reflect a greater tendency of MDAH-2774 cells to undergo necrotic cell death following treatment.

Overall, the obtained results confirm that Naph-3 exerts significant pro-apoptotic activitywith apoptosis induction observed in both IGROV-1 and MDAH-2774 cell lines. Although cisplatin demonstrated stronger pro-apoptotic efficacy, Naph-3 effectively promoted apoptotic cell death while maintaining relatively low necrotic activity, supporting its potential as a promising anticancer agent.

### 2.8. Impedance-Based Analysis of Cellular Response to Naph-3 and Cisplatin

**Figure 9:**
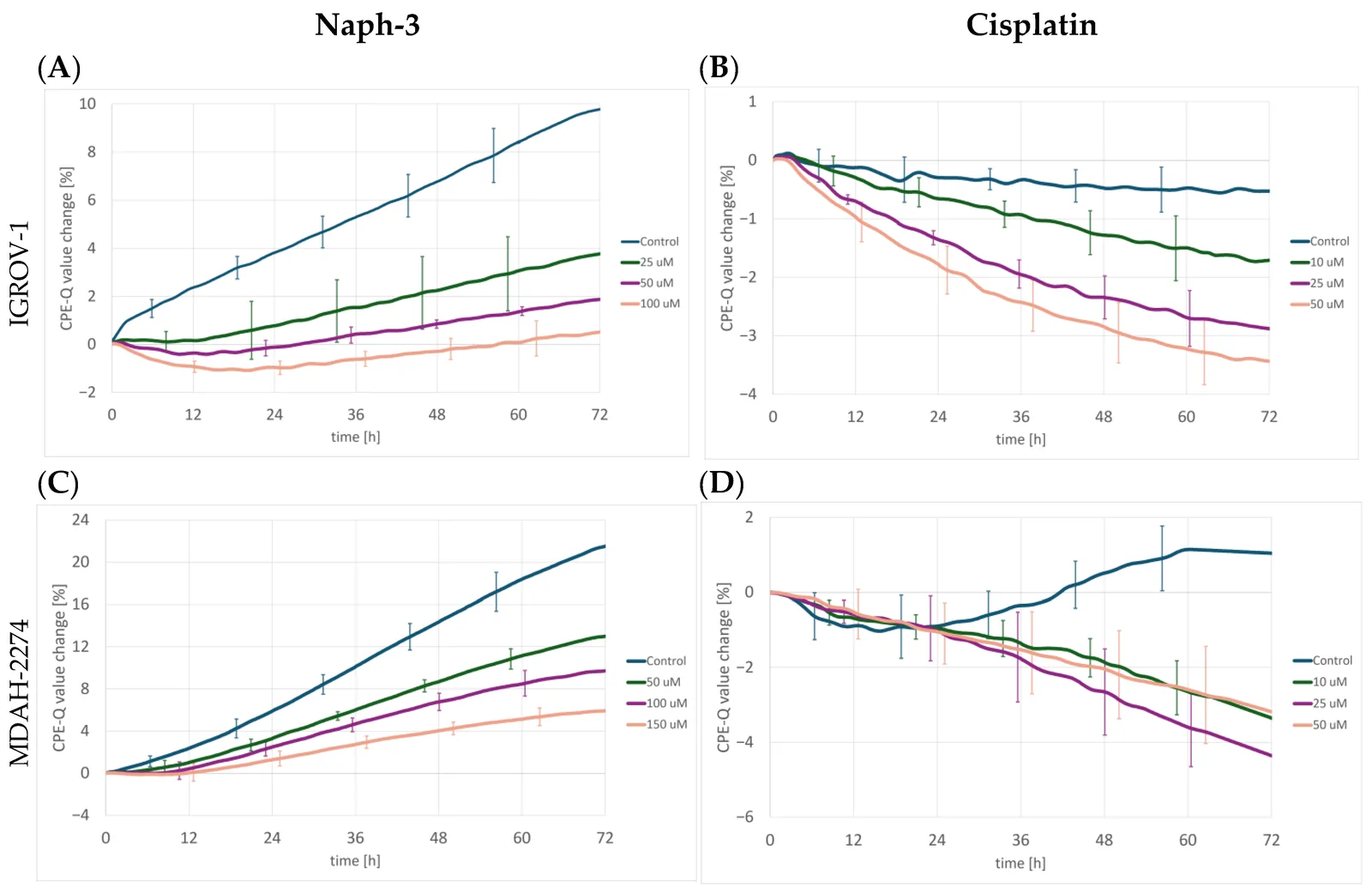

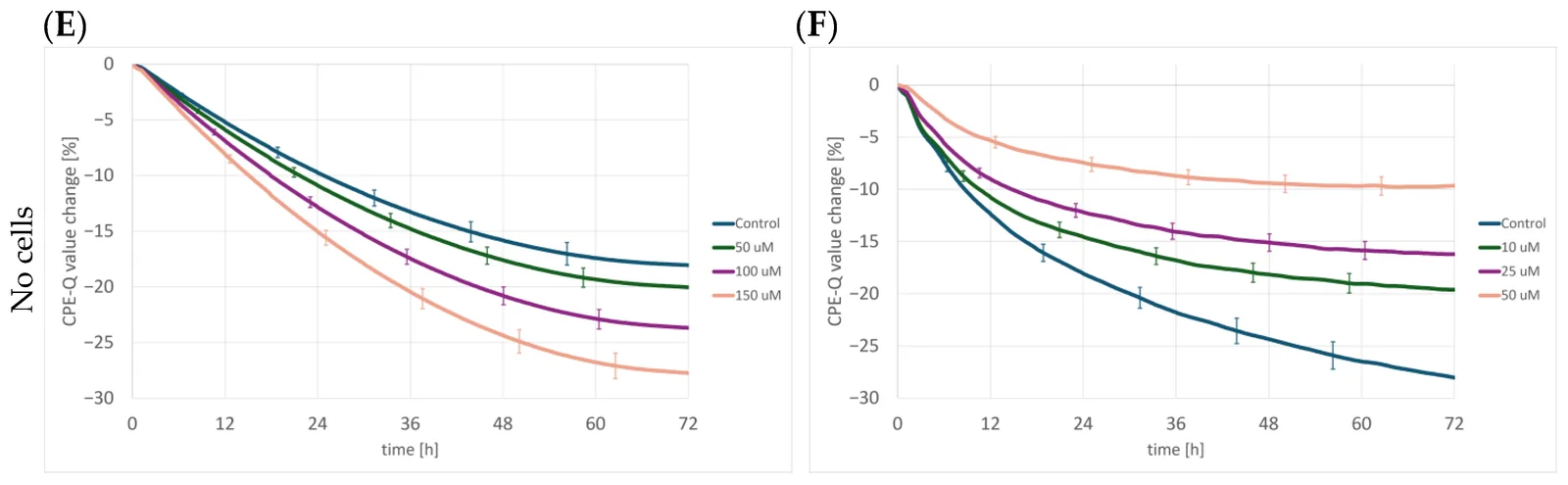
Time-dependent relative changes in the *CPE-Q* parameter in ovarian cancer cell cultures and cell-free controls exposed to Naph-3 or cisplatin. (**A**) IGROV-1 cells treated with cisplatin at 10, 25, and 50 µM; (**B**) IGROV-1 cells treated with Naph-3 at 25, 50, and 100 µM; (**C**) MDAH-2774 cells treated with cisplatin at 10, 25, and 50 µM; (**D**) MDAH-2774 cells treated with Naph-3 at 50, 100, and 150 µM; (**E**) cell-free controls containing culture medium alone or supplemented with cisplatin; and (**F**) cell-free controls containing culture medium alone or supplemented with Naph-3. For cell-containing samples, solid lines represent mean values, and shaded areas indicate standard deviations; cell-free plots show individual measurement traces. Untreated cell cultures and culture medium without cells served as the corresponding cellular and cell-free controls, respectively.

The impedance spectra were analyzed using the electrical equivalent circuit (EEC) modelling described in Section 4.11, and the fitted *CPE-Q* parameter was selected for time-course analysis. The measurements were done in series treated-untreated (Figure 9). In IGROV-1 cells culture, the untreated control showed a quasi-stable *CPE-Q* value over the analyzed time interval reaching −0.5% at the end. In contrast, cisplatin-treated cells culture demonstrated a gradual decrease in the *CPE-Q* parameter. After 72 h, the relative decrease in *CPE-Q* reached approximately −1.7%, −2.9%, and −3.4% for cisplatin concentrations of 10, 25, and 50 µM, respectively. This indicates that cisplatin affected the electrical response of the IGROV-1 medium-cell–electrode interface and shifted the *CPE-Q* parameter below the level observed in untreated cells.

A different response was observed for IGROV-1 cells culture treated with Naph-3. In this measurement series, the untreated control showed an increase in the *CPE-Q* parameter of approximately 9.8% after 72 h. In the presence of Naph-3, the *CPE-Q* parameter also increased over time, although the magnitude of this increase was lower than in the untreated control cell culture. After 72 h, the relative *CPE-Q* increase reached approximately 3.8%, 1.9%, and 0.5% for Naph-3 concentrations of 25, 50, and 100 µM, respectively. Thus, Naph-3 did not reverse the direction of *CPE-Q* changes in IGROV-1 cells but reduced the magnitude of the time-dependent increase in this parameter compared with untreated cells.

In MDAH-2774 cells culture, cisplatin induced a response opposite to that observed for the untreated control. The *CPE-Q* parameter in untreated cells increased by approximately 1.0% after 72 h, whereas cisplatin-treated cells showed a decrease in *CPE-Q*. The relative changes after 72 h were approximately −3.4%, −3.2%, and −4.3% for cisplatin concentrations of 10, 25, and 50 µM, respectively. No clear concentration-dependent trend was observed in this cell line; however, all cisplatin-treated samples showed a shift in the *CPE-Q* parameter below the initial reference level. These results indicate that cisplatin suppressed the positive time-dependent *CPE-Q* trend observed in untreated MDAH-2774 cells cultures.

In the MDAH-2774 cells culture series treated with Naph-3, the untreated control showed the strongest increase in *CPE-Q*, reaching approximately 22% after 72 h. In Naph-3-treated cells, the *CPE-Q* parameter also increased over time, but to a lower extent than in the untreated control. After 72 h, the relative increase in *CPE-Q* reached approximately 13.0%, 9.7%, and 5.9% for Naph-3 concentrations of 50, 100, and 150 µM, respectively. This indicates that Naph-3 modified the time-dependent electrical response of MDAH-2774 cells, although the direction of *CPE-Q* changes remained positive. Compared with IGROV-1 cells, the MDAH-2774 cells cultures exhibited a stronger positive drift of the *CPE-Q* parameter, in both untreated and Naph-3-treated cells cultures.

Cell-free controls were also analyzed to verify whether the observed *CPE-Q* changes could be attributed to the culture medium or to direct interactions between the tested compounds and the electrode surface. In wells containing medium alone, as well as medium supplemented with cisplatin or Naph-3, the *CPE-Q* parameter decreased over time. Therefore, the positive *CPE-Q* trend observed in untreated cell cultures and in Naph-3-treated cultures cannot be explained by the electrical behavior of the medium or the tested compounds alone. Instead, it indicates that the presence of adherent cells substantially modified the electrode interface or the medium properties and is counteracted by the decreasing *CPE-Q* trend observed in cell-free conditions. The increase in *CPE-Q* observed in cell-containing wells may result from progressive cell adhesion, spreading, electrode coverage, adsorption or secretion of biomolecules, and cell-induced changes in the local higher ionic concentration near the electrode surface. In contrast, in cell-free medium, the electrode–medium interface may undergo slow stabilization, adsorption, or degradation processes leading to a decrease in the *CPE-Q* parameter over time.

Overall, the *CPE-Q* parameter reflected compound- and cell-line-dependent changes in the measured systems. Cisplatin generally shifted *CPE-Q* values downward, producing either a decrease relative to the initial level or suppressing the natural increase observed in untreated controls. In contrast, Naph-3-treated cells retained a positive *CPE-Q* trend over time, although the magnitude of this increase was lower than in the corresponding untreated controls. These results suggest that the *CPE-Q* parameter should not be interpreted as a direct viability index, but rather as an integrated electrical descriptor of treatment-induced physicochemical changes in the medium-cell–electrode interface.

A discrepancy in *CPE-Q* trends was observed between untreated controls obtained in different experimental series. In principle, under identical experimental condition, untreated controls containing the same cells line cultured in the same culture medium should produce comparable *CPE-Q* trends. However, in IGROV-1 cells, the control cell culture from the cisplatin series was quasi-stable, whereas the control cell culture from the Naph-3 series increased by approximately 9.8% after 72 h. A similar difference was observed for MDAH-2774 cells line, where the untreated control cells culture increased by approximately 1.0% in the cisplatin series and by approximately 22.0% in the Naph-3 series. This discrepancy may result from series-to-series biological variability, including differences in initial cell seeding density, cell attachment and spreading, local electrode surface condition, evaporation or edge effects, and small differences in cell culture history between experimental runs. Therefore, untreated controls should be treated as series-specific references unless a common control plate or pooled control dataset is used.

## 3. Discussion

Naphthyridines constitute a structurally diverse class of heterocyclic compounds that display a broad spectrum of biological activities. Their anticancer potential has been extensively investigated over the years, with numerous studies demonstrating significant cytotoxic activity against a wide range of human cancer cell lines. Naphthyridine derivatives have been reported to inhibit the growth of various malignancies, including lung, breast, prostate, cervical, and liver cancers, as well as leukemia [9,14,15,16,18,21,27,31,32,33,34]. Importantly, a growing body of evidence supports the anticancer potential of naphthyridine derivatives in ovarian cancer, encompassing studies from initial in vitro screening assays through mechanistic investigations at the molecular level to advanced preclinical and clinical evaluations. Diego Rodríguez et al. synthetized novel 2,7-naphthyridine derivatives, which in preliminary screening exert significant cytotoxicity towards numerous cancer cell lines, including ovarian (IGROV-1, OVCAR-3, OVCAR-4, OVCAR-5, OVCAR-8, NCI/AD-RES, SKOV-3) with the average LC_50_ values in the range of 3.28-29.56 µM [25]. 1,6-naphthyridine derivative matrine was tested against ovarian cancer A2780 and SKOV3. The compound was shown to inhibit cell viability, migration, and invasion while simultaneously inducing apoptosis through reducing the phosphorylation levels of PI3K and Akt, downregulation of Bcl-2, and an increase in the expression of Bcl-xl. Importantly, the anticancer activity of matrine was also confirmed in vivo in an A2780 xenograft model, where it significantly suppressed tumor growth, increased apoptosis, and reduced tumor cell proliferation and angiogenesis, as indicated by decreased Ki-67 and CD31 staining. At the molecular level, matrine markedly reduced the phosphorylation of multiple cancer-associated signaling proteins, including ERK1/2, MEK1/2, PI3K, Akt, mTOR, FAK, RhoA, VEGFR2, and Tie2, without affecting their total expression levels. These in vivo findings were consistent with the molecular effects observed in vitro, supporting the involvement of multiple signaling pathways in the anticancer activity of matrine [22]. The effects in ovarian cancer models PA-1 and cisplatin-resistant SKOV-3 were also evaluated for isoaaptamine, a marine-derived naphthyridine alkaloid, which exerted potent anticancer activity as an apoptosis inducer through ROS generation, mitochondrial dysfunction, activation of caspases, and modulation of Bcl-2 family proteins [24]. As mentioned above, naphthyridine-based compounds have also found application in clinical investigations of ovarian cancer. Vosaroxin, a 1,8-naphthyridine topoisomerase II inhibitor, progressed to phase II clinical trials for cisplatin-resistant ovarian cancer patients. However, its clinical development was discontinued due to insufficient therapeutic efficacy [26].

In this research, three 2,7-naphthyridine derivatives Naph-1, Naph-2 and Naph-3 were tested against wide panel of cancer cell lines, including ovarian (IGROV-1, MDAH-2774 and SKOV-3), lung (A549), breast (MCF-7) and leukemia (K562). The cytotoxicity 2D-screening identified Naph-3 as the most promising derivative among the tested naphthyridines. The compound demonstrated activity against the majority of evaluated cancer cell lines, with the strongest effect observed in ovarian cancer models IGROV-1 and MDAH-2774. Interestingly, Naph-3 contains a 1,3,4-oxadiazole moiety at the 3-position of the naphthyridine ring. This distinguishes it structurally from the other derivatives investigated in this study: Naph-1 contains an ester group, while Naph-2 features an aromatic Schiff base structure at the 3-position. The 1,3,4-oxadiazole scaffold is a well-established pharmacophoric motif in medicinal chemistry, and naphthyridine–1,3,4-oxadiazole hybrids have previously demonstrated biological activity, including anti-cancer effects. In particular, 1,8-naphthyridine derivatives bearing a 1,3,4-oxadiazole moiety have been reported to exhibit anti-cancer activity and enhance the response of cancer cells to cisplatin [35,36]. Therefore, the presence of the 1,3,4-oxadiazole moiety in Naph-3 may contribute to its favourable activity profile observed in the present study. However, given the limited number of derivatives evaluated, further studies are required to determine its specific contribution to anti-cancer activity.

Although the cytotoxic potency of Naph-3 was lower than that observed for cisplatin, the compound exhibited a more favorable selectivity profile. The IC_50_ value obtained for the non-cancerous CHO-K1 cell line was substantially higher than those observed in ovarian cancer cells, indicating reduced toxicity toward normal cells. In contrast, cisplatin displayed comparable cytotoxicity in both cancerous and non-cancerous cells, which is consistent with its well-documented adverse effect profile. Therefore, while Naph-3 was less potent than the reference drug, its greater selectivity toward malignant cells may represent a significant advantage from a therapeutic perspective.

Although the anticancer activity of numerous naphthyridine derivatives has been extensively investigated in conventional monolayer cultures, studies evaluating their effects in three-dimensional tumor spheroid models remain scarce. Most published reports focused on cytotoxicity, apoptosis, and cell-cycle regulation in 2D systems, while data obtained in models more closely reflecting the in vivo tumor microenvironment are still limited [11,12,37]. Therefore, the present study extends the current knowledge on naphthyridine-based compounds by demonstrating the activity of Naph-3 in ovarian cancer spheroids and highlighting the differences between 2D and 3D treatment responses. The results obtained in IGROV-1 spheroids demonstrate that Naph-3 retains its anticancer activity in a three-dimensional tumor model despite the increased complexity and drug resistance typically associated with spheroid cultures. Notably, the highest tested concentration of Naph-3 produced a reduction in cell viability comparable to that achieved by cisplatin, indicating that the compound is capable of effectively penetrating and exerting cytotoxic effects within the spheroid structure. Interestingly, the results obtained in MDAH-2774 spheroids contrasted with those observed in conventional 2D cultures, where this cell line displayed high sensitivity to both cisplatin and Naph-3. The findings suggest that transition to a three-dimensional model profoundly altered the response of MDAH-2774 cells to treatment. This reduced sensitivity may be associated with characteristic features of mature spheroids, including restricted drug penetration, enhanced cell–cell interactions, and the presence of slowly proliferating or quiescent cell populations within the spheroid core [38,39,40]. Collectively, these results highlight the importance of incorporating 3D culture systems into anticancer drug evaluation and suggest that MDAH-2774 spheroids may represent a more clinically relevant, drug-resistant phenotype than that observed under conventional monolayer conditions.

The antiproliferative activity observed for Naph-3 in this study is consistent with previous reports describing naphthyridine derivatives as inhibitors of cancer cell growth. Numerous studies have demonstrated that the suppression of proliferation by naphthyridine-based compounds is frequently associated with cell cycle arrest and subsequent induction of apoptosis [10,12,41]. In study conducted by Rima D. Alharthy et al., pyrazolo-naphthyridine derivatives 5j and 5k induced cell cycle arrest at G0/G1 phase in HeLa and MCF-7 cells, with intercalative mode of binding in the compound-DNA interaction studies (comet assay) and increased levels of activated caspase-9 and -3/7 which confirmed activation of the intrinsic apoptotic pathway [12]. Treatment with the aaptamine derivative AP-51 resulted in cell cycle arrest at the G0/G1 phase, which was associated with alterations in cyclin-dependent kinase (CDK) expression. Furthermore, AP-51 triggered apoptosis via the intrinsic mitochondrial pathway, as reflected by nuclear fragmentation, suppression of the anti-apoptotic proteins BCL-XL and Mcl-1, and enhanced expression of cytochrome c, cleaved caspase-9, cleaved caspase-3, and cleaved PARP [41]. Mechanistic investigations of naphthyridine compounds 7 and 8 exerted their anticancer activity through dual inhibition of topoisomerases I and IIβ, resulting in G2/M cell cycle arrest and activation of the intrinsic apoptotic pathway accompanied by caspase-9 activation and downregulation of topoisomerase II expression [10]. These findings are in line with the results obtained in the present study, where Naph-3 induced G1-phase arrest and promoted apoptotic cell death in ovarian cancer cells, suggesting that modulation of cell cycle progression and activation of apoptosis may represent common mechanisms underlying the anticancer activity of naphthyridine derivatives. Naph-3 markedly reduced cell confluence in both ovarian cancer cell lines throughout the entire incubation period, indicating effective suppression of cell proliferation. This effect was accompanied by a accumulation of cells in the G1 phase and a reduction in the proportion of cells entering the S and G2/M phases, suggesting that inhibition of cell cycle progression contributes substantially to the observed antiproliferative activity. Furthermore, the induction of apoptosis detected after treatment with Naph-3 indicates that prolonged cell cycle arrest may ultimately trigger programmed cell death, thereby enhancing the overall anticancer effect of the compound. These results suggest that the antiproliferative activity of Naph-3 results from a combination of cell cycle inhibition and apoptosis induction, which is consistent with the mechanisms previously described for other biologically active naphthyridine derivatives [14,34,42]. Although Naph-3 induced G1 cell-cycle arrest and apoptosis, the molecular mechanisms underlying these effects remain to be elucidated. Further studies assessing key regulators of the G1/S transition, including cyclin D/CDK4/6, cyclin E/CDK2, p21, as well as apoptosis-related proteins and caspase activation, are required to determine the signaling pathways involved in Naph-3 activity. Therefore, the present findings should be considered as evidence of the cellular effects of Naph-3 rather than identification of its specific molecular targets.

In this study, the scratch assay was intended as a preliminary functional assessment of the potential effect of Naph-3 on the migration of IGROV-1 and MDAH-2774 ovarian cancer cells. These findings provide a basis for further studies using complementary migration-specific approaches to investigate the molecular mechanisms potentially involved in this effect, including EMT-related proteins and signalling pathways. The anti-migratory activity of Naph-3 observed in the present study is consistent with previous reports describing migration-inhibitory properties of selected naphthyridine derivatives. In ovarian cancer models PA-1 and SKOV-3, isoaaptamine have been shown to suppress adhesion, migration, invasion, and colony formation by ∼50%, suggesting that inhibition of tumor dissemination may represent an additional mechanism contributing to the anticancer activity of this class of compounds [24]. Xi Zhang et al. reported that matrine, quinolizidine alkaloid containing a naphthyridine-like scaffold, suppressed ovarian cancer cell A2780 and SKOV3 migration and invasion. This effect was associated with impaired formation of actin stress fibers and focal adhesions, accompanied by decreased activation of FAK and RhoA, suggesting disruption of signaling pathways involved in cancer cell motility [22]. Although the molecular basis of the anti-migratory effect of Naph-3 was not investigated in the present study, the significant reduction in wound closure observed in both ovarian cancer cell lines supports its potential to interfere with processes involved in metastatic progression. Notably, the anti-migratory effect of Naph-3 was comparable to that of cisplatin at selected concentrations, particularly in IGROV-1 cells, highlighting its potential as an effective inhibitor of ovarian cancer cell IGROV-1 and MDAH-2774 migration. These findings suggest that, in addition to its cytotoxic and pro-apoptotic properties, Naph-3 may contribute to limiting metastatic spread of ovarian cancer cells IGROV-1 and MDAH-2774.

Impedance spectroscopy provided a label-free and real-time readout of treatment-induced changes occurring within the medium–cell-electrode system. Unlike conventional endpoint assays, which characterize cellular status at a selected time point, impedance-based measurements enabled the progression of the cellular response to be followed throughout the incubation period. Impedance reflects an integrated response influenced by cell attachment, spreading, morphology, electrode coverage, cell–cell interactions, and other physicochemical changes in the environment. Accordingly, its biological interpretation requires comparison with independent assays of metabolic activity, confluence, apoptosis, cell-cycle distribution, and migration. Previous ECIS (electrical cell impedance sensing) studies have shown that impedance-based monitoring can detect dynamic responses to anticancer compounds and complement conventional endpoint assays [43,44,45]. Huang et al. monitored resistance and capacitance changes together with cell morphology and migration following cannabidiol and cisplatin treatment, whereas Liao et al. compared the impedance response with AlmarBlue, apoptosis, and wound-healing assays. The latter authors concluded that ECIS may reveal drug-induced effects earlier and in greater temporal detail than conventional biochemical measurements. Kim et al. similarly demonstrated an association between the capacitance-related signal, microscopic cell coverage, and the results of MTS and flow-cytometric analyses during drug-induced apoptosis. Zhang et al. reviewed electrical impedance sensing approaches and showed that impedance-derived parameters can reflect multiple cellular properties, including morphology, membrane integrity, growth state, and proliferation [46].

In contrast to aforementioned studies, based mainly on impedance or capacitance recorded at a selected frequency, the present analysis used complete impedance spectra and electrical equivalent circuit fitting (EEC). This allowed the time-dependent response to be assigned to the *CPE-Q* parameter, which described the capacitance-like behaviour of the medium-cell-electrode interface modified by adherent cells, while changes in bulk medium resistance were evaluated separately. Thus, the advantage of the present approach was a more explicit attribution of the measured response to a specific interfacial component. The fitted EEC contained only the elements that could be reliably distinguished in the measured spectra. A separate charge-transfer resistance was not identifiable and was therefore not included in the model, indicating that the measured response was dominated by interfacial polarization rather than by a distinct charge-transfer process.

Cisplatin generally caused a decrease in *CPE-Q* value, with a stronger effect at higher concentrations in IGROV-1 cells. This response was consistent with the marked reduction in confluence and increased apoptosis, as cell shrinkage, impaired adhesion, and detachment alter the electrical properties of the electrode interface. Naph-3 produced a different pattern: *CPE-Q* value remained positive but increased less strongly than in the untreated controls, particularly at higher concentrations. This suggests that cells continued to interact with and cover the electrode surface, although the development of the adherent cell layer was inhibited. The biological assays indicate that Naph-3 induced a mixed response involving both G1-phase cell-cycle arrest and apoptotic cell death, with a substantial cytostatic component, whereas cisplatin elicited a more pronounced cytotoxic response. This distinction was also reflected in the *CPE-Q* value kinetics. The positive but attenuated *CPE-Q* value trend observed after Naph-3 treatment was consistent with the continued presence of adherent cells despite suppressed proliferation and development of the cell layer. In contrast, the negative shift induced by cisplatin was consistent with more pronounced disruption and loss of the adherent cell layer. However, *CPE-Q* does not independently distinguish cytostatic from cytotoxic effects because it is also influenced by cell adhesion, morphology, spreading, and electrode coverage.

Cell-free controls showed a decrease in *CPE-Q* both in untreated medium and in medium containing the tested compounds. Therefore, the positive or less negative responses observed in cell-containing wells were associated mainly with the presence of adherent cells rather than with direct electrical effects of Naph-3 or cisplatin on the medium or electrodes. The differences between independent experimental series further indicate that quantitative interpretation should be based primarily on comparisons with the corresponding within-series controls.

The anticancer activity of Naph-3 appears to result from a combination of several complementary cellular effects. G1 cell-cycle arrest restricts proliferative capacity, while apoptosis induction contributes to reduced cancer cell survival. Inhibiting cell cycle progression may enhance cancer cells’ capacity to repair DNA damage, whereas irreparably damaged cells undergo apoptosis [47]. In parallel, inhibiting cell migration reduces their motile potential. Together, these findings suggest that Naph-3 affects several key cellular processes associated with the growth and progression of ovarian cancer in the IGROV-1 and MDAH-2774 cell lines, supporting its further investigation as a potential anticancer compound.

## 4. Materials and Methods

### 4.1. Synthesis and Structural Characterization

Three 2,7-naphthyridine derivatives were synthesized at the Department of Organic Chemistry and Pharmaceutical Technology of the Wroclaw Medical University. Commercially available chemicals for the synthesis were purchased from Chempur (Piekary Slaskie, Poland) or Sigma-Aldrich (Merck Group, Darmstadt, Germany) and were used without purification. The course of the reaction and the purity of the obtained compound were checked by thin-layer chromatography (TLC) using aluminium sheet Silica gel 60 F254 (Merck KGaA, Darmstadt, Germany) and visualized by ultraviolet light at 254 nm using CAMAG UV Lamp 4 (CAMAG, Muttenz, Switzerland). The melting point was determined by MEL-TEMP apparatus (Barnstead International, Dubuque, IA, USA) and was uncorrected.

FT-IR spectra were performed on the Thermo Scientific Nicolet iS50 FT-IR spectrometer (Thermo Fisher Scientific Inc., Waltham, MA, USA) using the ATR technique. FT-IR spectra analysis was performed using Omnic Spectra 2.0 software (Thermo Fisher Scientific Inc.).

^1^H and ^13^C NMR spectra were recorded in deuterated dimethyl sulfoxide (DMSO-*d*_6_) with TMS as the internal standard using Bruker NMR AVANCE III^TM^ 600 MHz spectrometer with Ascend^TM^ technology magnet (Bruker Corporation, Billerica, MA, USA). NMR spectral analyses were performer using MestReNova (Mnova version 14.2) software (Mestrelab Research S.L., Santiago de Compostela, Spain).

The FT-IR and elemental analyses were performed in the Laboratory of Elemental Analysis and Structural Research, Faculty of Pharmacy, Wroclaw Medical University.

Synthesis of 8-ethoxy-4-hydroxy-3-(1,3,4-oxadiazol-2-yl)-6-phenyl-2,7-naphthyridin-1(2H)-one (Naph-3):The mixture of 8-ethoxy-4-hydroxy-1-oxo-6-phenyl-1,2-dihydro-2,7-naphthyridine-3-carbohydrazide (0.01 mol) and triethyl orthoformate (0.02 mol) was refluxed with stirring for 6 h. After cooling, the precipitate was collected. Recrystallization from ethanol afforded the title compound Naph-3 as a beige solid in 63% yield; m.p. = 292–293 °C. Spectral analyses of the obtained compound are shown in the Supplementary Material (Figures S1–S3).

ATR-FTIR υ max (cm^−1^): 3160 (NH), 1644 (C=O), 1574 (C=N), 1303 (C-O).

^1^H NMR (600 MHz, DMSO-*d*_6_) δ (ppm): 1.44-1.46 (t, *J* = 6.0 Hz, 3H, CH_3_), 4.63–4.67 (q, *J* = 8.0 Hz, 2H, CH_2_), 7.50–7.58 (m, 3H, Ar), 7.91 (s, 1H, Ar), 8.22–8.23 (m, 2H, Ar), 8.36 (s, 1H, Ar), 11.60 (s, 1H, OH), 12.15 (s, 1H, NH).

^13^C NMR (600 MHz, DMSO-*d*_6_) δ (ppm): 14.36, 62.73, 103.84, 107.73, 111.38, 127.21 (2C), 128.50, 129.10 (2C), 130.67, 136.87, 138.96, 141.18, 152.37, 156.74, 160.46, 162.93.

Elemental Analysis calculated for formula C_18_H_14_N_4_O_4_: C, 61.71; H, 4.03; N, 15.99%; Found: C, 61.50; H, 4.25; N, 15.77%.

### 4.2. Preparation of Test Compounds

The 2,7-naphthyridine derivatives were dissolved in dimethylsulfoxide (DMSO, Sigma-Aldrich, St. Louis, MO, USA) as 20 mM stock solutions, and Cisplatin, purchased at Sigma-Aldrich, was dissolved in sterile water for injection. Stock solutions were stored at −20 °C. The working solutions were freshly prepared before each experiment by dilution of stock solutions in a complete culture medium to the desired final concentrations: 1, 10, 25, 50, 100 and 200 µM. The DMSO concentration in each solution did not exceed 1%.

### 4.3. Cell Lines and Conditions

The CHO-K1 (Chinese hamster ovary), SKOV-3 (human ovarian adenocarcinoma), K562 (human chronic myelogenous leukemia) and MDAH-2774 (human ovarian endometrioid adenocarcinoma) cell lines were obtained from the American Type Culture Collection (ATCC, Manassas, VA, USA). The IGROV-1 cell line was obtained from Merck Millipore (Darmstadt, Germany). The A549 (human alveolar basal epithelial adenocarcinoma) and MCF7 (human breast adenocarcinoma) cell lines were obtained from the European Collection of Authenticated Cell Cultures (ECACC, Salisbury, UK). The A549 and MCF-7 cell lines were grown in Minimum Essential Medium Eagle (EMEM) (Lonza, Verviers, Belgium). The IGROV-1 and K562 cell lines were cultured in Roswell Park Memorial Institute (RPMI) 1640 Medium (Lonza, Verviers, Belgium). The MDAH-2774 and SKOV-3 cells were cultured in Dulbecco’s Modified Eagle’s Medium (DMEM) (Lonza, Verviers, Belgium). The CHO-K1 cell line was cultured in Ham’s F-12 K Medium. Culture medias were supplemented with 10% fetal bovine serum (FBS) (Lonza, Verviers, Belgium), 25 μg/mL gentamicin (Lonza, Verviers, Belgium), and 2 mM L-glutamine (Lonza, Verviers, Belgium). The cell lines were incubated at temperature 37 °C with 5% CO2 and 95% humidity and subcultured twice a week using TrypLETMExpress solution (GIBCO, Waltham, MA, USA).

### 4.4. PrestoBlue Assay

The cytotoxicity of tested compounds was evaluated in PrestoBlue Assay. The tests were performed according to the manufacturer’s protocol. Briefly, the cells were seeded into 96-well culture plates and incubated for 24 h in a CO_2_ incubator. Subsequently, the culture medium was replaced with fresh medium containing the tested compounds, and the cells were incubated for an additional 72 h. Control cells were cultured in the absence of the tested compounds, both with and without DMSO addition. No significant differences in cell viability were observed between the control groups.

After the incubation period, PrestoBlue reagent (Thermo Fisher Scientific, Waltham, MA, USA) was added directly to each well according to the manufacturer’s recommendations, and the plates were incubated for 45 min in a CO_2_ incubator. Fluorescence was then measured using a Varioscan LUX microplate reader.

### 4.5. Spheroid 3D Models

The assessment of cell viability in 3D spheroid models were performed using 96-well U-bottom plates with a low-attachment surface. Cells were seeded at a density of 4000 cells per well. The plates were centrifuged at 2000× *g* rpm for 5 min and subsequently incubated for 6 days at 37 °C in a humidified atmosphere containing 5% CO_2_ to allow spheroid formation. Treatment with the tested compounds was performed on day 5.

After 72 h of incubation, Calcein-AM, Propidium Iodide and Hoechst 33342 from the Live Dead Cell Viability Assay Kit for 3D and 2D cell culture (Sigma-Aldrich, Darmstadt, Germany) were added to each well. Fluorescence intensity was measured using the Agilent BioTek Lionheart FX Automated Microscope (Agilent Technologies, Santa Clara, CA, USA) at excitation/emission wavelengths of approximately 469/525 nm for Calcein-AM, 531/593 nm for Propidium Iodide, 377/447 nm for Hoechst 33342.

### 4.6. Scratch Test

A scratch wound healing assay was performed according to a standard procedure to evaluate the effect of the tested compounds on tumor cell migration. Briefly, cells were seeded into 96-well plates containing fresh culture medium and incubated for 24 h in a CO_2_ incubator to allow cell attachment. A linear scratch was then created in the cell monolayer using a micropipette tip.

The selected compounds were added to the culture medium at final concentrations of 25, 50, and 100 µM for Naph-3 in the IGROV-1 cell line, 50, 100, and 150 µM for Naph-3 in the MDAH-2774 cell line, and 10, 25, and 50 µM for Cisplatin in both cell lines. Control cells were cultured in the absence of the tested compounds, both with and without DMSO addition. No differences in cell migration were observed between the control groups. Cell migration was monitored using the Lionheart FX automated live-cell imaging system (Agilent Technologies, Santa Clara, CA, USA). Images were acquired every 4 h for a total period of 72 h after scratching. The obtained images were analyzed using Gen5 software 3.17 (Agilent Technologies, Santa Clara, CA, USA).

### 4.7. Confluence Calculation

The effect of the tested compounds on cell proliferation was evaluated by real-time confluence analysis. Briefly, cells were seeded into culture plates and incubated with the tested compounds for 24 h. Control cells were cultured in the absence of the tested compounds, both with and without DMSO addition. No differences in cell proliferation were observed between the control groups.

Cell confluence was monitored using the Lionheart FX automated live-cell imaging system (Agilent Technologies, Santa Clara, CA, USA). The percentage of confluence was determined every 4 h for a total period of 72 h of the experiment.

### 4.8. Flow Cytometry

All samples were run on a Cyflow Space cytometer (Sysmex Partec GmbH, Görlitz, Germany) equipped with 488 nm, 638 nm, and 375 nm excitation lasers and four emission filters at 536/40, 590/50, 675/20, and 748 nm for 488 nm laser, 455/50 for 375 nm laser and 675/20 for 638 nm laser. The results were analyzed using FlowMax (Sysmex, Kobe, Prefektura Hyōgo, Japan) or FCS Express 4 flow software (De Novo Software, Glendale, CA, USA). The MultiCycle^TM^ DNA analysis model was used for cell cycle analysis. A dot plot gates were used on forward and side scatters to eliminate debris. Granulation, size, and fluorescence intensity were recorded for at least 20,000 cells per analysis.

### 4.9. Cell Cycle Analysis

Cell cycle distribution was analyzed by flow cytometry using the FxCycle^TM^ PI/RNase Staining Solution (Thermo Fisher Scientific, Waltham, MA, USA). Briefly, cancer cells were seeded into 6-well plates and incubated for 24 h under standard culture conditions. Subsequently, the culture medium was replaced with fresh medium containing the tested compounds, and the cells were further incubated for 72 h at 37 °C in a humidified atmosphere containing 5% CO_2_.

Following incubation, the cells were detached using TrypLE^TM^ Express (Gibco, Thermo Fisher Scientific, Grand Island, NY, USA) and washed twice with cold HBSS. The cells were then fixed in ice-cold 70% ethanol and stored on ice for at least 24 h prior to staining. Before analysis, the cells were washed with cold HBSS and resuspended in FxCycle^TM^ PI/RNase Staining Solution according to the manufacturer’s protocol. Samples were incubated for 30 min in the dark at room temperature and subsequently analyzed by flow cytometry.

Cell cycle distribution was determined based on DNA content, allowing quantification of the percentage of cells in the G0/G1, S, and G2/M phases using the MultiCycle^TM^ DNA analysis model.

### 4.10. Apoptosis Detection

Apoptosis was evaluated by flow cytometry using the Alexa Fluor^TM^ 488 Annexin V/Dead Cell Apoptosis Kit (Thermo Fisher Scientific, Waltham, MA, USA) according to the manufacturer’s protocol. Briefly, cancer cells were seeded into 6-well plates and incubated for 24 h under standard culture conditions. Subsequently, the culture medium was replaced with fresh medium containing the tested compounds, and the cells were further incubated for 72 h at 37 °C in a humidified atmosphere containing 5% CO_2_.

Following incubation, the cells were detached using Accutase^TM^ Cell Detachment Solution (Innovative Cell Technologies, Inc., San Diego, CA, USA), collected, and washed twice with cold PBS. The cell pellets were then resuspended in Annexin-binding buffer and stained with Alexa Fluor^TM^ 488 Annexin V and propidium iodide (PI). After incubation for 15 min in the dark at room temperature, the samples were immediately analyzed by flow cytometry. Based on fluorescence staining patterns, the cell populations were classified as viable cells (Annexin V−/PI−), early apoptotic cells (Annexin V+/PI−), late apoptotic cells (Annexin V+/PI+), and necrotic cells (Annexin V−/PI+).

### 4.11. Impedance Spectroscopy

Impedance-based monitoring was used as a label-free, time-resolved method to follow treatment-induced changes in cell behavior at the culture medium and the medium-cell–electrode interface. Similar impedance-based approaches using planar interdigitated electrodes and multichannel measurement platforms evaluated by electrical equivalent circuit modelling have previously been applied for real-time monitoring of biological surface-associated processes, including bacterial biofilm formation and degradation [48,49]. The use of impedance-based cell monitoring is also consistent with studies in adherent cancer cell models, where changes in impedance-derived parameters were associated with treatment-induced alterations in cell morphology, adhesion, viability, proliferation, apoptosis, and migration [43,44,46].

In the experiment xCELLigence E-plates L8 (Agilent Technologies, Santa Clara, CA, USA) were used as a culture platform. To enable impedance measurements the E-plate L8 contains integrated gold microelectrodes at the bottom of each well.

For impedance measurements, IGROV-1 and MDAH-2774 cells were seeded into E-plate L8 wells at a density of 1 × 10^4^ cells per well in 400 μL of complete culture medium. The tested compounds were added after 24 h of cell attachment at final concentrations of 10, 25, and 50 μM for cisplatin; 25, 50, and 100 μM for Naph-3 IGROV-1 series; and 50, 100, and 150 μM for Naph-3 MDAH-2774 series. Untreated cell cultures were used as cellular controls. Additional cell-free controls included culture medium alone and culture medium supplemented with cisplatin or Naph-3.

The prepared E-plate was mounted into the multiplexer socket and placed under standard cell culture conditions at 37 °C, 5% CO_2_, and 95% relative humidity. Impedance spectra were recorded continuously from 0 to 72 h in the frequency range from 1 Hz to 100 kHz using IMP-STM32 impedance analyser [50]. Each well was measured separately in real time in a quasi-simultaneous manner.

**Figure 10:**
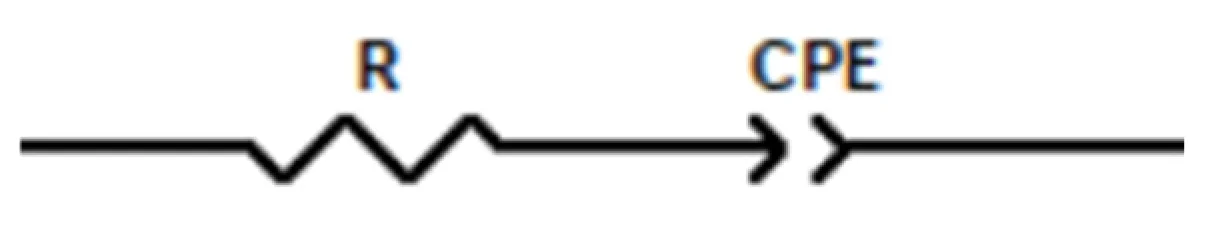
EEC of E-plate L8 single well.

The obtained impedance spectra were analyzed using electrical equivalent circuit (EEC) modelling with ZView software (Scribner Associates, Inc., Southern Pines, NC, USA). A simple EEC model (Figure 10) consisting of a resistor connected in series with a constant phase element (CPE) was used. The resistor represented the ohmic resistance of the culture medium, whereas the CPE represented the non-ideal interfacial response of the electrode–medium–cell system.

For each impedance spectrum, the EEC parameters were fitted and the *CPE-Q* parameter was extracted for further analysis. Time courses of the *CPE-Q* parameter were then processed to obtain relative percentage changes.

### 4.12. Statistical Analysis

Statistical analysis was performed using GraphPad Prism 8.0.1 software (GraphPad Software, La Jolla, CA, USA) and Microsoft Excel. Data normality was assessed using the Shapiro–Wilk and Kolmogorov–Smirnov tests. One-way ANOVA, Two-way ANOVA and two-way repeated measures ANOVA followed by Tukey’s post hoc test were used to evaluate differences between the tested compounds. Differences were considered statistically significant at *p* < 0.05.

## 5. Conclusions

Despite considerable advances in the management of ovarian cancer, the prognosis of patients with advanced disease remains poor due to late diagnosis, frequent recurrence, and the development of resistance to platinum-based chemotherapy. Therefore, the identification of novel compounds capable of targeting ovarian cancer cells through multiple mechanisms of action remains an important area of anticancer drug research. In this study, Naph-3 exhibited the most promising anticancer activity, demonstrating preferential cytotoxicity toward ovarian cancer cells IGROV-1 and MDAH-2774 while maintaining lower toxicity against non-cancerous cells compared with cisplatin. Mechanistic studies revealed that Naph-3 effectively inhibited cancer cell proliferation and migration, induced G1-phase cell cycle arrest, and promoted apoptotic cell death in both IGROV-1 and MDAH-2774 ovarian cancer models. Importantly, Naph-3 retained activity in IGROV-1 spheroids, highlighting its potential efficacy in a three-dimensional tumor environment that more closely resembles in vivo conditions. Although the overall anticancer potency of Naph-3 was lower than that of cisplatin, its favorable selectivity profile and multifaceted mechanism of action support its potential as a promising lead compound for further development. Impedance spectroscopy provided complementary kinetic information that was not available from individual endpoint assays. The CPE-Q parameter distinguished the different temporal responses induced by Naph-3 and cisplatin, supporting the interpretation of a substantial cytostatic component for Naph-3 and a more pronounced cytotoxic response to cisplatin. Nevertheless, the biological interpretation of CPE-Q remained dependent on validation using independent cellular assays. Future studies should focus on elucidating the molecular pathways responsible for its biological activity and evaluating its therapeutic efficacy in advanced preclinical models of ovarian cancer.

## Figures and Tables

**Table 1:** IC_50_ values (µM) of naphthyridine derivatives (Naph-1, Naph-2, and Naph-3) and cisplatin determined by the PrestoBlue cell viability assay after 72 h of treatment.

Cell Line	Naph-1	Naph-2	Naph-3	Cisplatin
IGROV-1	>200	>200	30.31 ± 2.63	9.50 ± 2.62
MDAH-2774	109.06 ± 4.99	152.90 ± 8.30	53.68 ± 11.20	11.54 ± 8.06
SKOV-3	161.43 ± 9.17	>200	>200	7.92 ± 5.76
MCF-7	171.06 ± 3.97	>200	159.17 ± 6.22	46.42 ± 9.27
A549	>200	>200	180.31 ± 7.74	21.57 ± 7.70
K562	>200	>200	50.52 ± 3.91	12.97 ± 6.32
CHO-K1	>200	>200	77.19 ± 7.95	10.50 ± 4.01

## Data Availability

The original contributions presented in this study are included in the article/Supplementary Material. Further inquiries can be directed to the corresponding authors.

## Supplementary Materials

The following supporting information can be downloaded at https://www.mdpi.com/article/10.3390/ph19101562/s1: Figure S1: FT-IR spectrum of compound Naph-3; Figure S2: ^1^H NMR spectrum of compound Naph-3; Figure S3: ^13^C NMR spectrum of compound Naph-3.
