# Supplementary material for: A Novel 2,7-Naphthyridine Derivative Suppresses Ovarian Cancer Cell Growth Through G1 Cell Cycle Arrest, Apoptosis Induction and Migration Inhibition
Source: Pharmaceuticals (Basel). 2026 Oct 1;19(10):1562. doi: 10.3390/ph19101562 (PMC13708021; doi:10.3390/ph19101562)
Supplement: Supplementary file 1 [file pharmaceuticals-19-01562-s001.zip › pharmaceuticals-4529855-supplementary.pdf]

SUPPLEMENTARY MATERIALS

A Novel 2,7-Naphthyridine Derivative Suppresses Ovarian Cancer Cell Growth Through G1 Cell Cycle Arrest, Apoptosis Induction and Migration Inhibition

Gabriela Chabowska [1,*], Konrad Chabowski [2], Helena Moreira [1], Anna Wójcicka [3], Anna Szyjka [1], Patrycja Koruba [4], Dorota Bęben [1], Tomasz Piasecki [5] and Ewa Barg [1,*]

[1] Department of Basic Medical Sciences, Faculty of Pharmacy, Wroclaw Medical University, Borowska Street 211, 50-556 Wroclaw, Poland; helena.moreira@umw.edu.pl (H.M.); anna.szyjka@umw.edu.pl (A.S.); dorota.beben@student.umw.edu.pl (D.B.)
[2] Independent Researcher, 52-131 Wroclaw, Poland; konrad.chabowski@gmail.com
[3] Department of Organic Chemistry and Pharmaceutical Technology, Faculty of Pharmacy, Wroclaw Medical University, Borowska 211A, 50-556 Wroclaw, Poland; anna.wojcicka@umw.edu.pl
[4] Faculty of Pharmacy, Wroclaw Medical University, Borowska Street 211, 50-556 Wroclaw, Poland; patrycja.koruba@student.umw.edu.pl
[5] Department of Nanometrology, Wroclaw University of Science and Technology, Z. Janiszewskiego 11/17, 50-372 Wroclaw, Poland; tomasz.piasecki@pwr.edu.pl
* Correspondence: gabriela.chabowska@student.umw.edu.pl (G.C.); ewa.barg@umw.edu.pl (E.B.); Tel.: +48-668719542 (G.C.); +48-717840477 (E.B.)

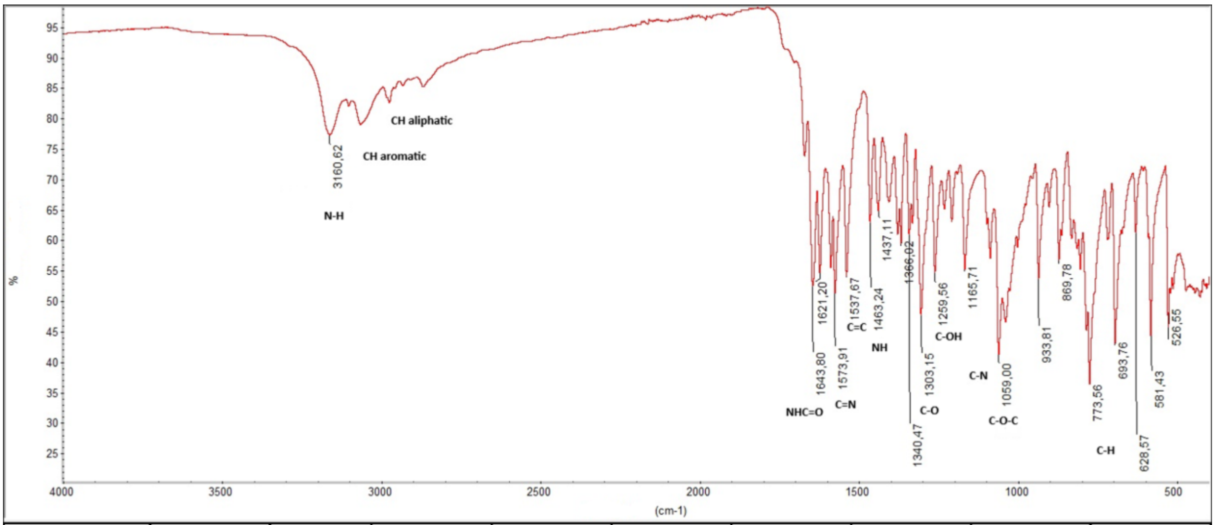

**Frequency (cm⁻¹)**	3160	2800-3000	1644	1574	1464	1303	1259	1165	1059
**Functional group**	NH	C-H	NHC=O	C=N	NH	C-O	C-OH	C-N	C-O-C

Figure S1. FT-IR spectrum of compound **Naph-3**.

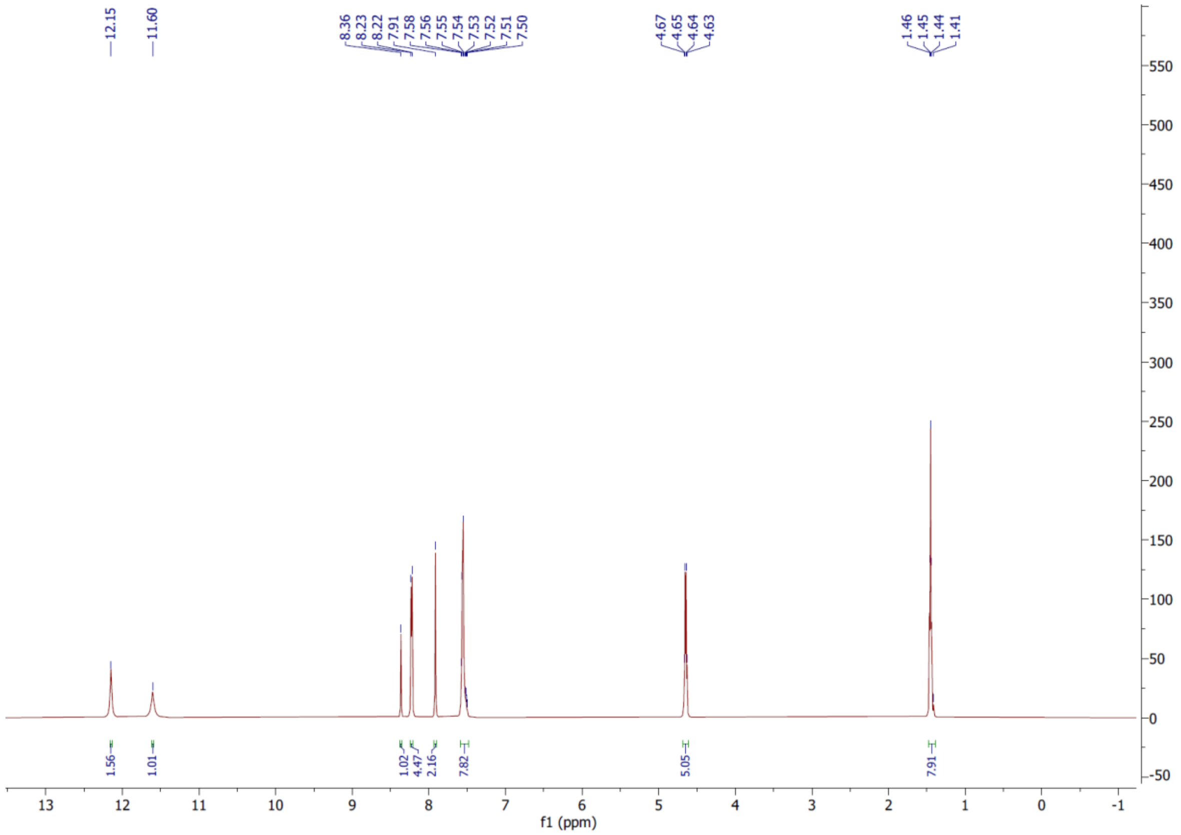

Figure S2. ^{1}H NMR spectrum of compound **Naph-3**.

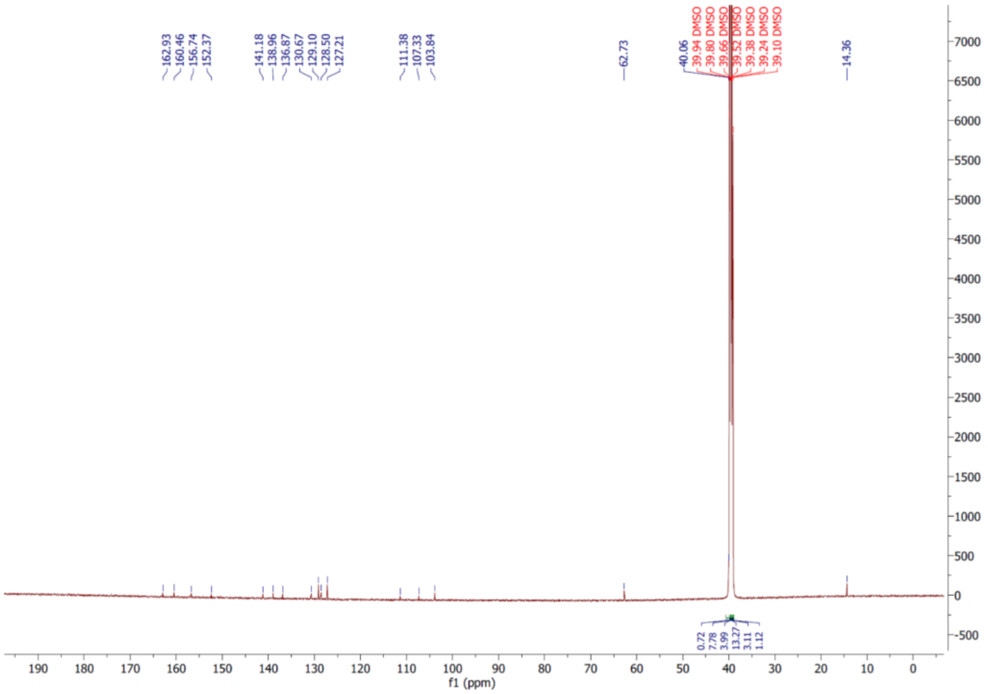

Delta	carbons
14.36	a
62.73	b
103.84	j
107.73	e
111.38	f
127.21	k
128.50	m
129.10	l
130.67	n
136.87	h
138.96	p
141.18	d
152.37	i
156.74	c
160.46	g
162.93	o

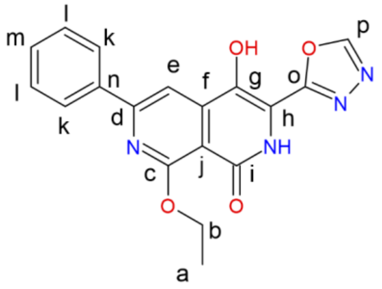

Figure S3. ^{13}C NMR spectrum of compound **Naph-3**.
